# Polymorphisms in the mTOR-PI3K-Akt pathway, energy balance-related exposures and colorectal cancer risk in the Netherlands Cohort Study

**DOI:** 10.1186/s13040-021-00286-3

**Published:** 2022-01-10

**Authors:** Colinda C.J.M. Simons, Leo J. Schouten, Roger W.L. Godschalk, Frederik-Jan van Schooten, Monika Stoll, Kristel Van Steen, Piet A. van den Brandt, Matty P. Weijenberg

**Affiliations:** 1 Department of Epidemiology, GROW – School for Oncology and Developmental Biology, Maastricht University, Maastricht, the Netherlands; 2 Department of Pharmacology and Toxicology, NUTRIM – School for Nutrition and Translational Research in Metabolism, Maastricht University, Maastricht, the Netherlands; 3 Institute of Human Genetics, Genetic Epidemiology, University of Münster, Münster, Germany; 4 Department of Biochemistry, Maastricht Centre for Systems Biology (MaCSBio), School for Cardiovascular Diseases, CARIM–, Maastricht University, Maastricht, the Netherlands; 5 GIGA-R Medical Genomics – BIO3, University of Liège, Liège, Belgium

**Keywords:** Body size, Cohort studies, Colorectal neoplasms, Mechanistic target of rapamycin, Polymorphisms

## Abstract

**Background:**

The mTOR-PI3K-Akt pathway influences cell metabolism and (malignant) cell growth. We generated sex-specific polygenic risk scores capturing natural variation in 7 out of 10 top-ranked genes in this pathway. We studied the scores directly and in interaction with energy balance-related factors (body mass index (BMI), trouser/skirt size, height, physical activity, and early life energy restriction) in relation to colorectal cancer (CRC) risk in the Netherlands Cohort Study (NLCS) (*n*=120,852). The NLCS has a case-cohort design and 20.3 years of follow-up. Participants completed a baseline questionnaire on diet and cancer in 1986 when 55–69 years old. ~75% of the cohort returned toenail clippings used for DNA isolation and genotyping (n subcohort=3,793, n cases=3,464). To generate the scores, the dataset was split in two and risk alleles were defined and weighted based on sex-specific associations with CRC risk in the other dataset half, because there were no SNPs in the top-ranked genes associated with CRC risk in previous genome-wide association studies at a significance level *p*<1*10^−5^.

**Results:**

Cox regression analyses showed positive associations between the sex-specific polygenic risk scores and colon but not rectal cancer risk in men and women, with hazard ratios for continuously modeled scores close to 1.10. There was no modifying effect observed of the scores on associations between the energy balance-related factors and CRC risk. However, BMI (in men), non-occupational physical activity (in women), and height (in men and women) were associated with the risk of CRC, in particular (proximal and distal) colon cancer, in the direction as expected in the lower tertiles of the sex-specific polygenic risk scores.

**Conclusions:**

Current data suggest that the mTOR-PI3K-Akt pathway may be involved in colon cancer development. This study thereby sheds more light on colon cancer etiology through use of genetic variation in the mTOR-PI3K-Akt pathway.

**Supplementary Information:**

The online version contains supplementary material available at 10.1186/s13040-021-00286-3.

## Introduction

Altered cell metabolism is considered a cancer hallmark associated with malignant cell growth. [1] Cell metabolism is influenced by the mammalian target of rapamycin (mTOR)-phosphatidylinositide 3-kinases (PI3K)-Akt pathway, which could therefore influence cancer development. In particular signaling by mTOR complex 1 (mTORC1) influences cell growth and survival via control of protein synthesis, autophagy, lipid synthesis, and mitochondrial metabolism. [2] Cellular energy status itself regulates mTOR-PI3K-Akt signaling, as do growth factors, stress, and nutrients. [2].

Genetic variation in the mTOR-PI3K-Akt pathway, which captures natural variation in the mTOR-PI3K-Akt pathway in the population, has been associated with cancer risk across organ sites. [3–12] Differences in associations between cancers may exist as, for example, *MTOR* rs2295080, a promotor variant associated with transcription [10] and mRNA expression [5] was oppositely associated with leukemia risk than with risk of other cancers. [7, 12, 13] To our knowledge, only one study investigated a potential interaction between *MTOR* rs2295080 and other variants in the mTOR-PI3K-Akt pathway and a diet risk score, showing evidence of interaction. [14].

Our aim was to extend on the existing evidence by studying mTOR-PI3K-Akt pathway genetic variation in relation to CRC risk and by investigating potential effect modification of mTOR-PI3K-Akt pathway genetic variation on associations between energy balance-related factors (body mass index, trouser/skirt size, height, physical activity, and early life energy restriction) and CRC risk in the large, prospective Netherlands Cohort Study. A higher body mass index, tallness, and a lack of physical activity are established CRC risk factors [15] which are thought to be associated with a positive energy balance and increased mTOR-PI3K-Akt signaling, stimulating malignant growth. Energy restriction during childhood and adolescence may favorably influence mTOR-PI3K-Akt signaling and could lower the potential for malignant growth. [2] Therefore, if we can show that the CRC risk conferred by these energy balance-related factors depends on genetic variation in the mTOR-PI3K-Akt pathway, which reflects core variation in the population, this provides evidence for that the mTOR-PI3K-Akt pathway is a mechanism that underlies associations between energy balance-related factors and CRC risk.

To achieve our aim, we generated sex-specific polygenic risk scores, capturing multiple polymorphisms in one variable. We generated the scores by splitting the dataset in two halves and only including polymorphisms which showed the same direction of association in relation to CRC risk in both datasets, as effect alleles could not be defined based on literature or existing genome-wide association studies (GWAS). We weighted the polymorphisms in the scores with the standard error weighted regression coefficients from the other set. The scores were then standardized and the scores and data were merged back together again, after which Cox hazard ratios for CRC were estimated for the scores (modeled in tertiles and continuously) and for the energy balance-related factors (modeled categorically) within tertiles of the scores.

## Results

### Baseline characteristics

**Fig. 1:**
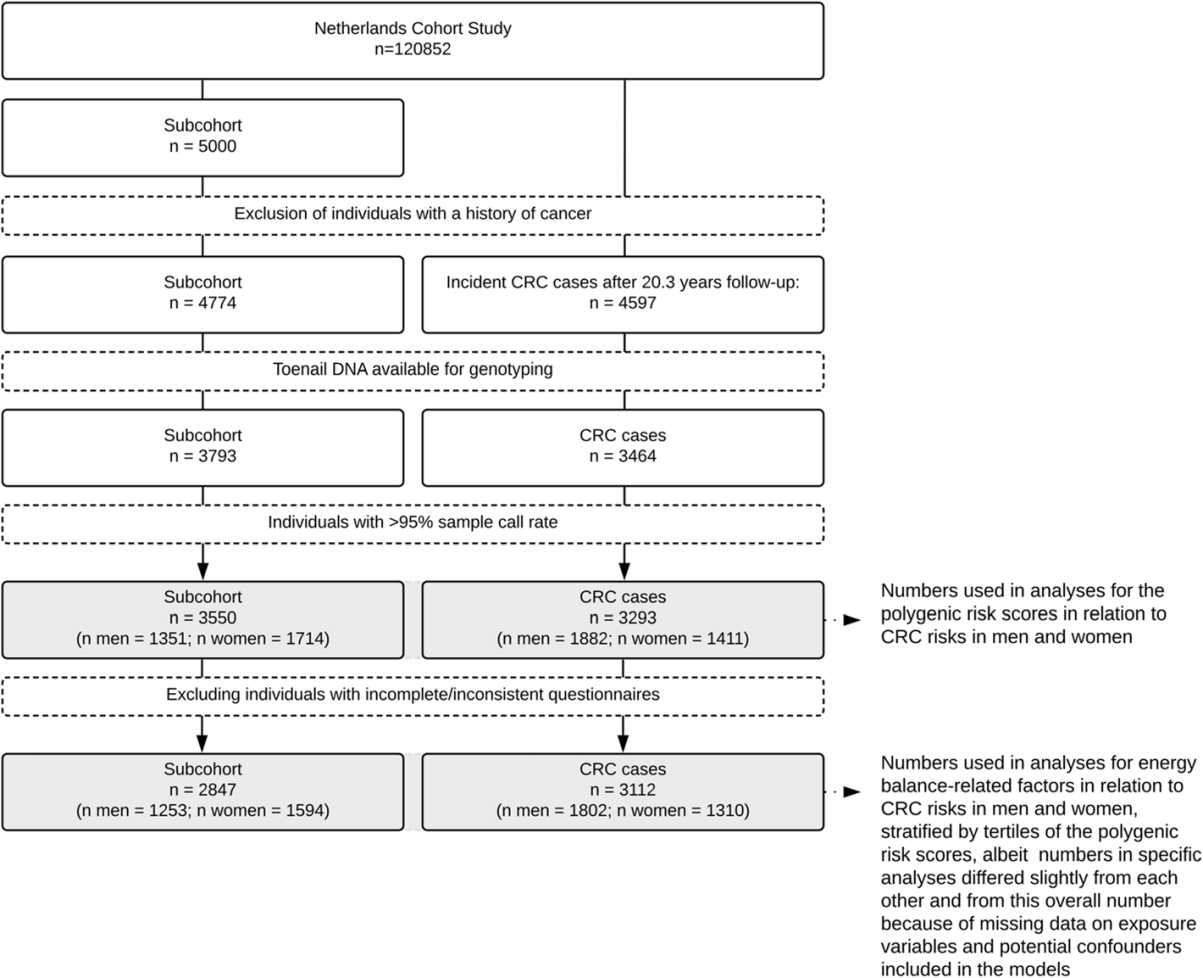
Flow diagram of subcohort members and colorectal cancer cases

**Fig. 2:**
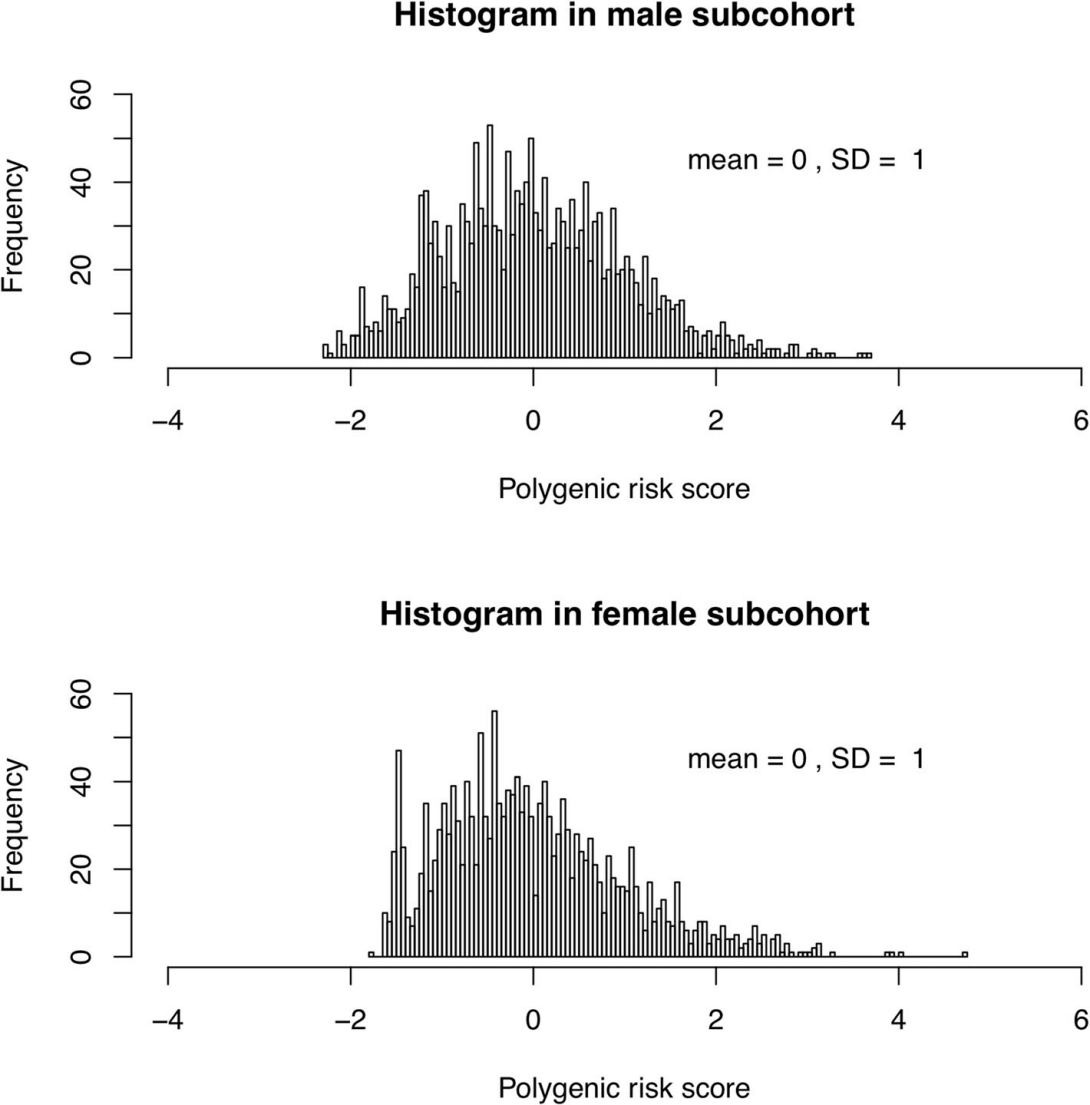
Histogram of the sex-specific polygenic risk scores in male and female subcohort members

A flow diagram leading up to the number of subcohort members and cases available in the NLCS for the current analyses is shown in Fig. 1. The polygenic risk score in men was made up of the following 12 SNPs out of a set of 24 genotyped SNPs in 10 top-ranked genes in the mTOR-PI3K-Akt pathway (Supplemental Tables 1 and 2): *MTOR* rs1057079, *TSC2* rs1800720, *TSC2* rs2516739, *PDK1* rs6723872, *EIF4EBP1* rs6605631, *RPS6KB2* rs12787021, *AKT3* rs14403, *AKT3* rs3006939, *AKT3* rs946824, *AKT2* rs16974157, *AKT2* rs874269, and *INSR* rs891088. The polygenic risk score in women was made up of the following 11 SNPs out of the 24 genotyped SNPs (Supplemental Tables 1 and 2): *MTOR* rs2295080, *TSC2* rs12918803, *PDK1* rs6723872, *RPS6KB2* rs12787021, *AKT3* rs1352162, *AKT3* rs14403, *AKT3* rs7523198, *AKT3* rs7523742, *AKT2* rs16974157, *AKT2* rs874269, and *INSR* rs891088. Supplemental Table 3 shows the regression coefficients, SEs, and resulting weights in the two dataset halves that were used to generate the sex-specific polygenic risk scores in each set. Supplemental Fig. 1 shows that the subcohort distributions of the standardized polygenic risk scores were similar in both sets. Since the scores were standardized, the mean equaled 0 and the SD equaled 1. The subcohort distributions of the sex-specific polygenic risk scores in the total population are shown in Fig. 2. The standardized score specific for men ranged from -2.25 to 3.70 in male subcohort members and from -2.25 to 3.55 in male CRC cases. The standardized score specific for women ranged from -1.79 to 4.70 in female subcohort members and from -1.65 to 4.54 in female CRC cases. Table 1 shows that the mean scores within tertiles were comparable between subcohort members and CRC cases in both men and women. Table 1 furthermore shows baseline characteristics of the NLCS cohort, with no major differences in the distributions of most baseline characteristics between subcohort members and CRC cases in men and women. The most notable difference between subcohort members and CRC cases was in the percentage of first-degree family history of CRC (men: 8.8% versus 5.3%, respectively; women: 9.4% versus 5.5%, respectively).

**Table 1:** Baseline characteristics of subcohort members and CRC cases within the Netherlands Cohort Study (20.3 years of follow-up)

	Men				Women			
	**Subcohort**		**CRC cases**		**Subcohort**		**CRC cases**	
**Characteristic**	**N (%)**	**Mean (SD)**	**N (%)**	**Mean (SD)**	**N (%)**	**Mean (SD)**	**N (%)**	**Mean (SD)**
Polygenic risk score ^a^								
Tertile 1		-1.06 (0.4)		-1.02 (0.4)		-1.01 (0.3)		-0.99 (0.3)
Tertile 2		-0.06 (0.3)		-0.05 (0.3)		-0.13 (0.2)		-0.12 (0.2)
Tertile 3		1.12 (0.6)		1.15 (0.6)		1.14 (0.7)		1.17 (0.7)
Age in years ^b^		61.4 (4.2)		61.7 (4.1)		61.6 (4.3)		62.0 (4.1)
BMI at baseline, kg/m^2^ (sex-specific) ^b^								
Tertile 1		22.3 (1.4)		22.5 (1.2)		21.6 (1.4)		21.6 (1.4)
Tertile 2		24.8 (0.5)		24.9 (0.6)		24.7 (0.8)		24.8 (0.8)
Tertile 3		27.9 (1.7)		27.9 (2.0)		29.1 (2.7)		29.0 (2.6)
Non-occupational physical activity, min/d ^b^								
<=30	322 (17.8)		308 (16.6)		432 (25.6)		387 (27.9)	
>30-60	576 (31.8)		556 (29.9)		541 (32.1)		433 (31.2)	
>60-90	915 (50.5)		993 (53.5)		712 (42.3)		569 (41.0)	
Height, cm (sex-specific) ^b^								
Tertile 1		169.4 (3.3)		169.2 (3.4)		158.9 (3.7)		159.1 (3.2)
Tertile 2		176.4 (1.6)		176.5 (1.5)		166.2 (1.5)		166.3 (1.5)
Tertile 3		184.0 (3.8)		184.1 (4.1)		172.4 (3.1)		172.9 (3.5)
Residence during the Hunger Winter (1944-45) ^b^								
Western city	903 (60.4)		962 (61.9)		911 (56.8)		725 (54.8)	
Western rural area	228 (15.3)		232 (14.9)		250 (15.6)		223 (16.9)	
Non-western area	364 (24.3)		360 (23.2)		442 (27.6)		375 (28.3)	
Family history of CRC, yes ^b^	98 (5.3)		166 (8.8)		94 (5.5)		133 (9.4)	
Smoking status								
Never	235 (12.1)		228 (12.1)		1010 (59.0)		844 (60.0)	
Ex-smoker	957 (52.2)		1100 (58.5)		349 (20.4)		303 (21.5)	
Current smoker	642 (35.0)		551 (29.3)		352 (20.6)		261 (18.5)	
Alcohol intake, g/d ^b^								
0	250 (13.9)		227 (12.3)		512 (32.3)		416 (31.3)	
0.1-29	1278 (71.1)		1315 (71.2)		1018 (64.2)		856 (64.4)	
≥30	270 (15.0)		304 (16.5)		55 (3.5)		58 (4.4)	
Meat intake, g/d ^b^		104.7 (43.4)		105.4 (43.0)		92.5 (41.8)		89.6 (40.7)
Processed meat intake, g/d ^b^		16.6 (17.5)		17.4 (17.2)		10.7 (12.4)		10.8 (11.6)
Total energy intake, kcal/d ^b^		2140 (505)		2147 (496)		1658 (411)		1650 (388)

Abbreviations: BMI, body mass index; CRC, colorectal cancer; N, number of; SD, standard deviation

^a^ The polygenic risk score was composed of *MTOR* rs1057079, *TSC2* rs1800720, *TSC2* rs2516739, *PDK1* rs6723872, *EIF4EBP1* rs6605631, *RPS6KB2* rs12787021, *AKT3* rs14403, *AKT3* rs3006939, *AKT3* rs946824, *AKT2* rs16974157, *AKT2* rs874269, and *INSR* rs891088 in men, and of *MTOR* rs2295080, *TSC2* rs12918803, *PDK1* rs6723872, *RPS6KB2* rs12787021, *AKT3* rs1352162, *AKT3* rs14403, *AKT3* rs7523198, *AKT3* rs7523742, *AKT2* rs16974157, *AKT2* rs874269, and *INSR* rs891088 in women. Scores were standardized with a mean of 0 and an SD of 1.

^b^ Numbers and percentages or means and SDs were given after additionally excluding individuals with incomplete/inconsistent questionnaires.

### Sex-specific polygenic risk scores of mTOR-PI3K-Akt polymorphisms and CRC risk

Positive associations were observed between the sex-specific polygenic risk scores and CRC risk when modeling these in tertiles and continuously (Table 2). Men had a 7% increase in CRC risk per unit increase on the polygenic risk score specific for men (HR_continuous_ = 1.07, 95% CI: 1.00-1.15; HR_tertile 2 vs. 1_ = 1.07, 95% CI: 0.91-1.26; HR_tertile 3 vs. 1_ = 1.14, 95% CI: 0.97-1.35). Women had a 9% increase in CRC risk per unit increase on the polygenic risk score specific for women (HR_continuous_ = 1.09, 95% CI: 1.01-1.17; HR_tertile 2 vs. 1_ = 0.97, 95% CI: 0.81-1.16; HR_tertile 3 vs. 1_ = 1.15, 95% CI: 0.97-1.38). Similar positive (borderline) statistically significant associations were observed for colon cancer risk and proximal and distal colon cancer risk in men and women. The associations between the polygenic risk scores and rectal cancer risk in men and women were positive in direction, but not statistically significant.

**Table 2:** Polygenic risk scores of mTOR-PI3K-Akt pathway polymorphisms in relation to CRC risk by sex and subsite in the Netherlands Cohort Study after 20.3 years of follow-up

			Men			Women				
**Endpoint**	**Polygenic risk score**^**a**^	**PT at risk**	**N cases**	**HR**^**b**^	**(95% CI)**	**PT at risk**	**N cases**	**HR**^**b**^	**(95% CI)**	
CRC	T2 vs. T1	9796 vs. 9540	633 vs. 587	1.07	(0.91,1.26)	10,066 vs. 10,207	434 vs. 460	0.97	(0.81,1.16)	
	T3 vs. T1	9506 vs. 9540	662 vs. 587	1.14	(0.97,1.35)	10,125 vs. 10,207	517 vs. 460	1.15	(0.97,1.38)	
	Continuous	28,841	1882	1.07	(1.00,1.15)	30,397	1411	1.09	(1.01,1.17)	
Colon	T2 vs. T1	9796 vs. 9540	397 vs. 376	1.05	(0.87,1.27)	10,066 vs. 10,207	312 vs. 337	0.95	(0.78,1.16)	
	T3 vs. T1	9506 vs. 9540	444 vs. 376	1.20	(1.00,1.44)	10,125 vs. 10,207	395 vs. 337	1.21	(1.00,1.46)	
	Continuous	28,841	1217	1.10	(1.03,1.19)	30,397	1044	1.11	(1.03,1.20)	
Proximal colon	T2 vs. T1	9796 vs. 9540	177 vs. 175	1.02	(0.80,1.30)	10,066 vs. 10,207	185 vs. 200	0.96	(0.76,1.21)	
	T3 vs. T1	9506 vs. 9540	213 vs. 175	1.24	(0.98,1.57)	10,125 vs. 10,207	232 vs. 200	1.20	(0.96,1.51)	
	Continuous	28,841	565	1.11	(1.01,1.22)	30,397	617	1.09	(0.99,1.20)	
Distal colon	T2 vs. T1	9796 vs. 9540	206 vs. 190	1.07	(0.85,1.35)	10,066 vs. 10,207	120 vs. 129	0.95	(0.72,1.25)	
	T3 vs. T1	9506 vs. 9540	222 vs. 190	1.18	(0.94,1.49)	10,125 vs. 10,207	153 vs. 129	1.21	(0.93,1.58)	
	Continuous	28,841	618	1.11	(1.02,1.22)	30,397	402	1.14	(1.02,1.27)	
Rectum	T2 vs. T1	9796 vs. 9540	162 vs. 159	1.00	(0.78,1.29)	10,066 vs. 10,207	88 vs. 85	1.06	(0.77,1.47)	
	T3 vs. T1	9506 vs. 9540	163 vs. 159	1.04	(0.81,1.33)	10,125 vs. 10,207	87 vs. 85	1.05	(0.76,1.44)	
	Continuous	28,841	484	1.00	(0.91,1.11)	30,397	260	1.07	(0.94,1.22)	

Abbreviations: CRC, colorectal cancer; CI, confidence interval; HR, hazard ratio; PT, person-time; T1-3, sex-specific tertile 1-3; vs. versus.

^a^ The polygenic risk score was composed of *MTOR* rs1057079, *TSC2* rs1800720, *TSC2* rs2516739, *PDK1* rs6723872, *EIF4EBP1* rs6605631, *RPS6KB2* rs12787021, *AKT3* rs14403, *AKT3* rs3006939, *AKT3* rs946824, *AKT2* rs16974157, *AKT2* rs874269, and *INSR* rs891088 in men, and of *MTOR* rs2295080, *TSC2* rs12918803, *PDK1* rs6723872, *RPS6KB2* rs12787021, *AKT3* rs1352162, *AKT3* rs14403, *AKT3* rs7523198, *AKT3* rs7523742, *AKT2* rs16974157, *AKT2* rs874269, and *INSR* rs891088 in women.

^b^ Adjusted for age (years).

Individual SNP-CRC risk associations are shown in Supplemental Table 4. Several statistically significant associations were observed between individual SNPs, predominantly *AKT3* SNPs, and CRC risk in men and women after gene-based FDR adjustment.

### Energy balance-related exposures and CRC risk: effect modification by sex-specific polygenic risk scores of mTOR-PI3K-Akt polymorphisms?

Table 3 shows the associations between BMI, trouser/skirt size, BMI at age 20, non-occupational physical activity, height, and energy restriction during childhood and adolescence and CRC risk in men and women, stratified by tertiles of the sex-specific polygenic risk scores. BMI was positively associated with CRC risk in men in the lowest tertile of the polygenic risk score specific for men; non-occupational physical activity was inversely associated with CRC risk in women in the lowest tertile of the polygenic risk score specific for women; and height was positively associated with CRC risk in men and women in the middle tertile of the polygenic risk score specific for each sex and in the lowest tertile of the polygenic risk score specific for women. No significant multiplicative interactions were observed. Analyses for subsite-specific CRC risks are shown in Supplemental Tables 5-8. In these stratified analyses for subsite-specific CRC risks, BMI was positively associated with proximal colon cancer risk in men, height was positively associated with colon, proximal colon, and distal colon cancer risk in both men and women and with rectal cancer risk in women, and non-occupational physical activity was inversely associated with colon, proximal colon, and distal colon cancer risk, with most associations observed in the lower tertiles of the polygenic risk score specific for each sex. Furthermore, one statistically significant interaction was observed between energy restriction during childhood and adolescence and the polygenic risk score specific for men in relation to distal colon cancer risk. Exposure to energy restriction during childhood and adolescence was inversely associated with distal colon cancer risk in men in the middle tertile of the polygenic risk score specific for men, while the association in the lowest tertile was positive in direction, though not statistically significant, nor was the association in the highest tertile statistically significant.

**Table 3:** Associations between exposures related to energy balance and CRC risk in men and women, stratified for tertiles of the sex-specific polygenic risk score of mTOR-PI3K-Akt pathway polymorphisms in the Netherlands Cohort Study (20.3 years of follow-up)

		Men										Women									
		**BMI**										**BMI**									
		**T1 (sex-specific)**			**T2 (sex-specific)**			**T3 (sex-specific)**				**T1 (sex-specific)**			**T2 (sex-specific)**			**T3 (sex-specific)**			
		**N cases/PT at risk**	**HR**^**a,b**^	**(95% CI)**	**N cases/PT at risk**	**HR**^**a,b**^	**(95% CI)**	**N cases/PT at risk**	**HR**^**a,b**^	**(95% CI)**	**P for interaction**	**N cases/PT at risk**	**HR**^**a,b**^	**(95% CI)**	**N cases/PT at risk**	**HR**^**a,b**^	**(95% CI)**	**N cases/PT at risk**	**HR**^**a,b**^	**(95% CI)**	**P for interaction**
**Polygenic risk score**	**T1**	165/3343	1.00	(ref.)	166/2911	1.07	(0.78,1.46)	203/2588	1.48	(1.08,2.04)		133/3207	1.00	(ref.)	141/2999	1.16	(0.83,1.62)	118/2788	1.03	(0.72,1.47)	
	**T2**	165/2907	1.00	(ref.)	207/3196	1.12	(0.82,1.52)	206/2810	1.26	(0.91,1.76)		139/2829	1.00	(ref.)	132/2822	0.98	(0.69,1.38)	109/2775	0.81	(0.55,1.17)	
	**T3**	186/2688	1.00	(ref.)	201/3046	0.93	(0.68,1.27)	219/2726	1.07	(0.77,1.47)	0.34	157/3000	1.00	(ref.)	137/2900	0.86	(0.62,1.20)	151/2746	1.04	(0.74,1.44)	0.54
		**Trouser/skirt size**										**Trouser/skirt size**									
		**≤median (sex-specific)**			**>median (sex-specific)**							**≤median (sex-specific)**			**>median (sex-specific)**						
		**N cases/PT at risk**	**HR**^**a,c**^	**(95% CI)**	**N cases/PT at risk**	**HR**^**a,c**^	**(95% CI)**				**P for interaction**	**N cases/PT at risk**	**HR**^**a,c**^	**(95% CI)**	**N cases/PT at risk**	**HR**^**a,c**^	**(95% CI)**				**P for interaction**
**Polygenic risk score**	**T1**	163/3250	1.00	(ref.)	321/5042	1.12	(0.81,1.54)					168/3967	1.00	(ref.)	220/4910	1.00	(0.69,1.45)				
	**T2**	176/2932	1.00	(ref.)	359/5019	1.17	(0.86,1.60)					176/3849	1.00	(ref.)	198/4437	1.03	(0.70,1.50)				
	**T3**	177/3062	1.00	(ref.)	376/4775	1.25	(0.92,1.71)				0.84	178/3628	1.00	(ref.)	263/4937	1.11	(0.79,1.56)				0.76
		**Non-occupational physical activity**										**Non-occupational physical activity**									
		**≤30 min/day**			**>30-60 min/day**			**>60 min/day**				**≤30 min/day**			**>30-60 min/day**			**>60 min/day**			
		**N cases/PT at risk**	**HR**^**a,d**^	**(95% CI)**	**N cases/PT at risk**	**HR**^**a,d**^	**(95% CI)**	**N cases/PT at risk**	**HR**^**a,d**^	**(95% CI)**	**P for interaction**	**N cases/PT at risk**	**HR**^**a,d**^	**(95% CI)**	**N cases/PT at risk**	**HR**^**a,d**^	**(95% CI)**	**N cases/PT at risk**	**HR**^**a,d**^	**(95% CI)**	**P for interaction**
**Polygenic risk score**	**T1**	89/1380	1.00	(ref.)	156/3011	0.79	(0.54,1.17)	289/4452	1.01	(0.70,1.45)		113/1887	1.00	(ref.)	104/2964	0.57	(0.39,0.84)	175/4142	0.70	(0.49,0.99)	
	**T2**	83/1378	1.00	(ref.)	177/2811	1.09	(0.74,1.62)	318/4724	1.14	(0.80,1.65)		98/1775	1.00	(ref.)	128/2790	0.84	(0.57,1.24)	154/3860	0.72	(0.50,1.04)	
	**T3**	102/1341	1.00	(ref.)	182/2766	0.78	(0.53,1.16)	322/4353	0.86	(0.60,1.23)	0.66	102/1933	1.00	(ref.)	157/2815	1.10	(0.76,1.59)	186/3898	0.91	(0.64,1.29)	0.67
		**Height**, **T1 (sex-specific)**			**Height**, **T2 (sex-specific)**			**Height**, **T3 (sex-specific)**				**Height**, **T1 (sex-specific)**			**Height**, **T2 (sex-specific)**			**Height**, **T3 (sex-specific)**			
		**N cases/PT at risk**	**HR**^**a,c**^	**(95% CI)**	**N cases/PT at risk**	**HR**^**a,c**^	**(95% CI)**	**N cases/PT at risk**	**HR**^**a,c**^	**(95% CI)**	**P for interaction**	**N cases/PT at risk**	**HR**^**a,c**^	**(95% CI)**	**N cases/PT at risk**	**HR**^**a,c**^	**(95% CI)**	**N cases/PT at risk**	**HR**^**a,c**^	**(95% CI)**	**P for interaction**
**Polygenic risk score**	**T1**	169/3074	1.00	(ref.)	182/2862	1.24	(0.90,1.71)	183/2906	1.24	(0.91,1.71)		126/3605	1.00	(ref.)	144/3156	1.34	(0.96,1.88)	122/2233	1.70	(1.18,2.44)	
	**T2**	185/3205	1.00	(ref.)	189/2616	1.44	(1.04,1.98)	204/3093	1.30	(0.95,1.77)		133/3130	1.00	(ref.)	131/2905	1.00	(0.71,1.42)	116/2390	1.05	(0.72,1.52)	
	**T3**	181/2826	1.00	(ref.)	205/2987	1.14	(0.83,1.56)	220/2647	1.28	(0.93,1.76)	0.94	138/3285	1.00	(ref.)	162/2761	1.60	(1.13,2.26)	145/2600	1.57	(1.09,2.27)	0.30
		**Exposure to energy restriction during childhood and adolescence as based on place of residence during the Hunger Winter**										**Exposure to energy restriction during childhood and adolescence as based on place of residence during the Hunger Winter**									
		**Non-Western area**			**Western rural area**			**Western city**				**Non-Western area**			**Western rural area**			**Western city**			
		**N cases/PT at risk**	**HR**^**a,c**^	**(95% CI)**	**N cases/PT at risk**	**HR**^**a,c**^	**(95% CI)**	**N cases/PT at risk**	**HR**^**a,c**^	**(95% CI)**	**P for interaction**	**N cases/PT at risk**	**HR**^**a,c**^	**(95% CI)**	**N cases/PT at risk**	**HR**^**a,c**^	**(95% CI)**	**N cases/PT at risk**	**HR**^**a,c**^	**(95% CI)**	**P for interaction**
**Polygenic risk score**	**T1**	269/4418	1.00	(ref.)	73/1127	1.24	(0.82,1.86)	107/1918	0.98	(0.70,1.38)		201/4989	1.00	(ref.)	60/1182	1.42	(0.92,2.19)	108/2291	1.24	(0.88,1.75)	
	**T2**	305/4307	1.00	(ref.)	69/1230	0.85	(0.57,1.27)	112/1696	0.84	(0.60,1.19)		194/4413	1.00	(ref.)	63/1222	1.20	(0.79,1.82)	101/2333	1.02	(0.72,1.43)	
	**T3**	324/4194	1.00	(ref.)	74/946	1.15	(0.76,1.74)	108/1805	0.72	(0.50,1.02)	0.56	225/4412	1.00	(ref.)	70/1283	1.12	(0.75,1.67)	127/2462	1.04	(0.75,1.43)	0.61

Abbreviations: BMI, body mass index; CI, confidence interval; HR, hazard ratio; N, number of; PT, person-time; ref., reference; T1-3, tertile 1-3.

^a^ Adjusted for age (years), first-degree family history of colorectal cancer (yes/no); smoking status (never, ex, current); alcohol intake (0, 0.1-29, ≥30 g/d); meat intake (g/d), processed meat intake (g/d), and total energy intake (kcal/d).

^b^ Additionally adjusted for non-occupational physical activity (≤30, >30-60, >60 min/day), respectively.

^c^ Additionally adjusted for BMI (kg/m^2^) and non-occupational physical activity (≤30, >30-60, >60 min/day), respectively.

^d^ Additionally adjusted for BMI (kg/m^2^).

## Discussion

The associations observed between the sex-specific polygenic risk scores and the risk of CRC overall, specifically colon cancer risk, suggest that the mTOR-PI3K-Akt pathway is involved in colon cancer development in both men and women. Involvement of the mTOR-PI3K-Akt pathway in rectal cancer development cannot be concluded based on the current data. There were no (multiplicative) interactions between the energy balance-related exposures studied and the polygenic risk scores specific for each sex in relation to CRC risk overall or by subsite, except for one, i.e. there was an interaction with energy restriction during childhood and adolescence in relation to distal colon cancer risk in men. However, associations within tertiles of the polygenic risk score did not provide a clear indication for a modifying effect. Overall, in the stratified analyses, we predominantly observed associations between energy balance-related exposures and CRC risk in the lower tertiles of the sex-specific polygenic risk scores, with the direction of the associations generally in line with what would be expected for these factors in relation to CRC risk based on literature [15] and with what was previously observed in the NLCS after 16.3 years of follow-up. [16, 17] However, these stratum-specific associations on their own, without (statistical) interaction present, do not form sufficient evidence for concluding that there was a modifying effect by mTOR-PI3K-Akt genetic variation on associations between energy balance-related factors and CRC risk. That said, if we allow for speculation and view these data in a broader sense, these data raise the question whether environmental factors predominate when genetic risk is low.

As regards to our findings for the polygenic risk scores and subsite-specific CRC risks, stronger involvement of the mTOR-PI3K-Akt pathway in the development of more proximally located colorectal tumors is plausible considering that higher (over)expression of Akt1, Akt2, and p-p70S6K(Thr389) genes has been reported in proximal colon tumors than distal colon tumors. [18] PTEN gene expression was also found to show a positive expression gradient towards the proximal colon, starting at the rectum. [19] Furthermore, *PTEN* and *PIK3CA* mutations are more prevalent in tumors of the proximal colon. [20] These literature findings provide some confidence in that the associations observed in this study, which suggest mTOR-PI3K-Akt involvement in colon cancer development, were not chance findings.

There are few data characterizing associations between energy balance-related exposures and CRC risk within genetic risk strata based on mTOR-PI3K-Akt pathway polymorphisms or *vice versa*. We specifically chose the former modulation in light of future translation of the results towards prevention, because polymorphisms are static variables and energy balance-related exposures such as BMI are modifiable; that is, a healthy BMI and physical activity level could be especially important for specific genetic risk groups. Previous studies, however, investigated cancer risks associated with carrying more risk alleles within strata of energy balance-related factors, [21–24] under the hypothesis that a positive energy imbalance activates the mTOR-PI3K-Akt pathway. These studies did not uniformly suggest that activation of the mTOR-PI3K-Akt pathway by a positive energy imbalance influences cancer risk, as some observed associations between mTOR-PI3K-Akt pathway variants and cancer risk in normal weight instead of overweight/obese individuals. [23] Meanwhile, energy balance has been shown to modulate signaling through Akt and mTOR in multiple epithelial tissues in mice, with diet-induced obesity enhancing and calorie restriction inhibiting activation. [25] The mixed observational results in the literature might be explained by differences in effect on CRC risk of the specific variants included, perhaps suggesting the importance of capturing a sufficient and representative amount of genetic variation present in the mTOR-PI3k-Akt pathway in the population. For example, one of the studies referenced above utilized both a polygenic risk score of mTOR-PI3K-Akt pathway polymorphisms and an energy balance index and found a joint effect of the two on bladder cancer risk. [21] This study, however, may have had limited power, leading to unstable (and extreme) risk estimates, as based on the case numbers and the wide confidence intervals reported. In addition, this study selected SNPs for inclusion in the risk score based on p-values for main effects and tested the risk score in the same population as in which the single SNPs were tested, which might have led to overfitting of the risk score model to the underlying data and inflation of the results. Alternatively, the mixed results in the literature in relation to CRC risk and the absence of interaction in the present study could mean that an interaction between energy balance-related exposures and genetic variation in the mTOR-PI3K-Akt pathway in relation to CRC risk is absent or not strong enough to be detected given the average statistical power achieved in a large observational cohort.

Despite the absence of (statistical) interaction between energy balance-related exposures and the polygenic risk score of mTOR-PI3K-Akt pathway polymorphisms in relation to CRC risk, one particular finding in this study is noteworthy. This is the observation that height was a colon cancer risk factor in both men and women in the lowest and middle tertiles of the polygenic risk score. Previously, after 16.3 years of follow-up, height was observed to be a colon cancer risk factor in women but not men, [16] whereas accounting for genetic variation in the mTOR-PI3K-Akt pathway appeared to remove the sex difference observed overall in our cohort. We have observed the same phenomenon when accounting for genetic variation in the insulin-like growth factor pathway. [26] The absence of a sex difference is in accordance with the literature that shows energy balance-related exposures such as BMI and height to be CRC risk factors regardless of sex. [15] Interestingly, BMI and height were colon but not rectal cancer risk factors in this study and in previous studies from the NLCS regardless of which other variables were taken into account, [16, 26] which may be a cohort-specific effect (e.g. residual confounding in this specific population), as the literature shows these factors to also be rectal cancer risk factors. [15].

The methodology used to select genes and polymorphisms in the mTOR-PI3K-Akt pathway and the methodology used to generate the sex-specific weighted polygenic risk scores of mTOR-PI3K-Akt polymorphisms deserves some further discussion. Firstly, the assumptions made to select key genes in the mTOR-PI3K-Akt pathway using the relative betweenness centrality measure may not accurately represent the biology of the mTOR-PI3K-Akt pathway. For example, it was assumed that the information flow (signals) between nodes (genes) in a pathway is undividable and always takes the shortest path. In addition, we have assumed an undirected graph (pathway), meaning the information flow between connected nodes can go both ways. These assumptions were nevertheless necessary and resulted in a list of top-ranked genes that fit with prior knowledge of key players in the mTOR-PI3K-Akt pathway, reassuring us that no major bias occurred because of a potentially inaccurate representation of the biology of the pathway. Secondly, our method of SNP selection, i.e. we selected tagging variants in order to cover as much of the genetic variation in the top-ranked genes as possible, did not immediately allow us to consider correlations of SNPs with other biological levels, such as gene or protein expression. Many selected SNPs, however, turned out to be expression quantitative trait loci (eQTLs) for the gene that they were tagging and/or other genes according to the Genotype-Tissue expression (GTex) project (https://gtexportal.org/home/; National Institutes of Health, United States). Thirdly, we were limited in the number of SNPs that we could genotype, and thus the number of genes in the mTOR-PI3K-Akt pathway that we could cover, because of budgetary constraints that allowed us to genotype only one multiplex assay. Given the genes that we covered, this may have led to insufficient coverage of genes encoding for proteins of which signaling is under the influence of a negative energy imbalance. For example, we could not include SNPs encoding for adenosine monophosphate-activated protein kinase (AMPK), which phosphorylates TSC2 in the TSC1-2 complex [2] and stabilizes the mTOR-RAPTOR bond in mTORC1 under conditions of a negative energy balance, inhibiting mTORC1 signaling. [2, 27].

Strengths of this study include that it is a large, population-based prospective cohort with long follow-up, resulting in a large number of CRC cases and making selection and information bias unlikely. Limitations include the single baseline measurement of exposures. The NLCS population has been found stable in its dietary habits, [28] but diminishing physical activity levels and changes in body composition may be inevitable with increasing age, possibly having led to attenuation of associations over time.

## Conclusions

The findings of this study suggest that the mTOR-PI3K-Akt pathway may be involved in the development of colon cancer, but not rectal cancer. Energy balance-related factors were associated with CRC risk as hypothesized, mostly within the lower tertiles of the polygenic risk score specific for each sex, but there was no clear modifying effect of the scores. The relevance of this study lies in its contribution to the evidence base on mechanisms involved in colon cancer development through use of a polygenic risk score, capturing natural variation in the mTOR-PI3K-Akt pathway in the population.

## Methods

### Population and design

The NLCS [29] includes 120,856 men and women who completed a questionnaire on diet and cancer at baseline in 1986 when 55-69 years old. The baseline questionnaire included a 150-item semi-quantitative food frequency questionnaire, which was found to rank individuals’ dietary intake adequately as compared to a 9-day dietary record [30] and was shown a good indicator of intake for at least 5 years. [28] Approximately 75% of the cohort returned toenail clippings, which are a valid and long-term DNA source. [31, 32] The NLCS is characterized by a case-cohort approach for reasons of efficiency related to questionnaire processing, follow-up, and genotyping. A random subcohort (*n*=5000), selected immediately after baseline and independent of any exposure, is followed up for vital status through record linkage to the Central Bureau of Genealogy and municipal population registries (>99.9% completeness) to estimate the accumulated person-time at risk. Participants were excluded if they reported a history of cancer other than skin cancer at baseline, leaving 4774 subcohort members for follow-up (Fig. 1). The whole cohort is followed up for incident cancer cases through record linkage to the population-based cancer registry and PALGA (the Netherlands pathology database) (>96%completeness). [33, 34] The case-cohort design allows for the estimation of hazard ratios as would be done in a full cohort under the assumption that the fraction of the accumulated person-time at risk observed for exposed and unexposed individuals is equal. This is reasonable considering that the subcohort was selected independent of any exposure. The extra variance introduced by sampling the subcohort from the total cohort can be adjusted for using the robust variance estimator. [35] A detailed description of the NLCS is available in [29]. After 20.3 years of follow-up from September 1986 until the end of 2006, there were 3144 incident colon cancer cases (ICD-O-3 code C19) (among which 1623 incident proximal colon cancer cases (ICD-O-3 codes C18-C18.4) and 1430 incident distal colon cancer cases (ICD-O-3 codes C18.5-C18.7)), 427 incident rectosigmoid cancer cases (ICD-O-3 code C20), and 1026 incident rectal cancer cases (ICD-O-3 code C21), totaling to 4597 incident CRC cases (Fig. 1).

### Baseline information

Baseline information included height (cm) and weight (kg) used to derive body mass index (kg/m^2^) (BMI) (reflecting body fatness), trouser/skirt size (Dutch clothing sizes) which is used as a marker for waist circumference (reflecting abdominal fatness when adjusting for BMI), weight at age 20 used together with height to derive BMI at age 20 (kg/m^2^), and energy restriction during childhood and adolescence as based on place of residence during the Dutch Hunger Winter in 1944-45. Self-reports on weight and height have been shown valid measures in other cohort studies with >10 years of follow-up. [36, 37] Trouser/skirt size correlated with hip and waist circumferences in a subset of weight-stable NLCS men (r=0.63 and 0.64, respectively) and women (r=0.78 and 0.71, respectively) and was associated with endometrial and renal cancer risk in a fashion as would be expected for waist circumference. [38] BMI and height measures were divided into sex-specific tertiles based on the distribution in the subcohort. Trouser/skirt size was split into two sex-specific categories based on the median in the subcohort. Information on non-occupational physical activity in categories of ≤30, 30-60, >60 min of physical activity per day was a sum measure of daily walking/cycling (min/day), weekly recreational walking/cycling, weekly gardening/doing odd jobs, and weekly sports/gymnastics (categories: never, 1, 1-2, >2 h/week). More details on energy restriction during childhood and adolescence as measured in the NLCS are available in [39]. Baseline information on relevant covariates in diet and lifestyle was also available from the baseline questionnaire.

### DNA isolation and genotyping

Toenail clippings were stored without further treatment or climate control of the storage room. The DNA isolation protocol has been described in [31] and [32]. DNA isolated from toenail material was stored at -30 °C at the BioBank Maastricht University Medical Center+ (Maastricht, the Netherlands). Toenail DNA is suitable for genotyping on the Agena BioScience MassARRAY® platform (Hamburg, Germany), allowing the genotyping of 36-40 SNPs at once, although, in practice, not all SNPs can be combined due to sequence incompatibilities between the sequences flanking the SNPs.

### Gene and SNP selection

We identified 10 top ranked genes in the mTOR-PI3K-Akt pathway according to their relative betweenness centrality, which provides an indication of the strength of node involvement in the information flow through a network: *MTOR* (alias: *FRAP1*), *TSC2*, *PDPK1 (*alias: *PDK1)*, *EIF4EBP1* (alias: *4EBP1*), *IRS1*, *RPS6KB1 (*alias: *S6K1)*, *RPS6KB2 (*alias: *S6K2)*, *AKT3*, *AKT1*, and *AKT2* (Supplemental Table 1). The Kyoto Ecyclopedia of Genes and Genomes (KEGG) mTOR signaling (map04150) was used as input (http://www.genome.jp/kegg/) (R software, version 3.2.2, KeggGraph package). Since there were no SNPs in these genes associated with colorectal cancer risk at a significance level of *p*<1*10^−5^ in GWAS (https://www.ebi.ac.uk/gwas/), we selected tagging single nucleotide polymorphisms (tagSNPs) at a minor allele frequency of 5% or higher for the top 10 ranked genes using aggressive tagging [40] (HaploView version 4.2, Broad Institute). Not all 10 genes could be included in the assay because not all combinations of SNPs can be included because of sequence incompatibilities between the sequences flanking the SNPs on the basis of which the primer design took place. We firstly fixed the replicated cancer risk-associated *MTOR* SNP rs2295080 in the assay design. The assay design next allowed for the inclusion of the following tagSNPs covering 7 of the 10 top-ranked genes: *MTOR* rs1057079; *TSC2* rs2516739, rs1800720, rs2074969, rs9928737, and rs12918803; *PDK1* rs6723872; *EIF4EBP1* rs6605631; *RPS6KB2* rs12787021; *AKT3* rs3006939 rs14403, rs7523198, rs7523742, rs1352162, and rs946824; and *AKT2* rs874269, rs16974157, and rs7250897. The assay was furthermore filled up as much as possible with single genome-wide association study (GWAS) hits for anthropometric traits, physical activity, or CRC annotated to mTOR-PI3K-Akt pathway genes (https://www.ebi.ac.uk/gwas/). Included were *RPTOR* rs7503807 for its association with obesity, [41] *RICTOR* rs2043112 and *S6K1* rs1051424 for their association with (childhood) obesity-associated traits, [42] and *IGF1R* rs2871865 [43, 44] and *INSR* rs891088 for their association with height (abbreviations are explained in Supplemental Table 1). [43, 45].

### Genotyping

Genotyping was performed for 3793 (79.5%) subcohort members and 3464 (75.3%) CRC cases with available toenail DNA (Fig. 1). Potentially contaminated samples as noted by the laboratory technicians were excluded (2.6%) to ensure the quality of the data. Mean sample call rates were 97.4% (median: 100%). SNP call rates were between 97 and 99%, except for one SNP, which had a SNP call rate of 92% (rs1051424). A sample call rate of 95% or higher was present in samples from 3550 subcohort members (93.6%) and in samples from 3293 CRC cases (95.1%). (Two SNPs genotyped for use in another project were also enumerated when calculating the sample call rate.) Allele frequencies in the subcohort, which is representative of the whole cohort, are given in Supplemental Table 2. Hardy-Weinberg Equilibrium was violated on five occasions, but we did not exclude these SNPs from further analysis, because we had no reason to suspect genotyping errors since all SNPs were genotyped using a single assay and because multiple tests increased the risk of a significant finding by chance.

### Statistical analysis

The main exposure variable used in the analyses was a sex-specific polygenic risk score. Since no GWAS summary statistics were available to generate a polygenic risk score, we generated this score using the data at hand. First, the dataset was divided into two random sets of approximately equal size (datasets A and B). In each set, each individual SNP was modelled continuously in relation to the risk of CRC in men and women separately, adjusting the model for age. We deemed it important to do this in a sex-specific manner, because energy balance-related risk factors for CRC have been shown to differ between men and women in the NLCS. [16, 17, 26, 39, 46–48] Specifically, a larger BMI and trouser size, used as a proxy for waist circumference, were previously shown to be risk factors in men but not women, whereas height was a risk factor in women but not men in the NLCS. Genotypes in individuals were coded ‘0’ when homozygote for the major allele, ‘1’ when heterozygote, and ‘2’ when homozygote for the minor allele. We used the standard error (SE) weighted regression coefficients (beta / SE) from set A to generate the polygenic risk scores in set B and *vice versa* (i.e. two polygenic risk scores were generated in each set, one for men and one for women). The polygenic risk scores were calculated by weighting the number of risk alleles carried by an individual in one set using the standard error weighted regression coefficient from the other set (SNP x: n risk alleles * (beta / SE), with n being 0, 1, or 2) and then summing the weighted number of risk alleles for all SNPs into a single score. In case of a negative standard error weighted regression coefficient, the coding of the SNP was reversed, as in these instances the major allele instead of the minor allele was considered the risk-conferring allele, and the absolute value of the weighted regression coefficient was used. We only included SNPs in the polygenic risk scores that showed the same direction of effect in both sets so that the risk scores in each dataset would include the same SNPs, though different weights were used to generate the scores. The scores were allowed to include different SNPs between men and women. We also only included SNPs in the polygenic risk scores that were in low linkage disequilibrium (LD) which was defined as r^2^≤0.6, because SNPs in low LD are most likely to add new information to the score in terms of the variance explained in the outcome. LD was evaluated for the data under study using default settings in Haploview version 4.2 and defining CRC cases as affected individuals and subcohort members without CRC as unaffected individuals. There were two pairs of SNPs that were in LD with r^2^>0.6 (*AKT3* rs946824 and *AKT3* rs7523742 and *MTOR* rs2295080 and *MTOR* rs1057079), of which only one of a pair (chosen at random) was included in the polygenic risk score in case of consistent betas in sets A and B for both SNPs. To adjust for missing SNP data (one SNP was missing at most because of exclusion of samples with <95% call rate), we divided the polygenic risk scores in each set by the proportion of successfully genotyped risk alleles [(n successfully genotyped SNPs*2) / (n genotyped SNPs*2)]. We then standardized the set-specific polygenic risk scores by deducting the mean and dividing the scores by their standard deviation (SD) [(x-mean) / SD], with sex-specific means and SDs based on the subcohort. This allowed us to merge the scores and the datasets back together again, resulting in one dataset which included two polygenic risk scores, i.e. one for men and one for women.

Cox regression was then used to study the (subsite-specific) CRC risks associated with the sex-specific polygenic risk scores in men and women separately using R (R software, version 3.2.2). Models were age-adjusted and the polygenic risk scores were modelled in tertiles (based on the distribution in the male and female subcohort, respectively) and continuously. We also analyzed individual SNPs in relation to CRC risks in men and women in the total dataset, assuming a codominant and additive inheritance mode, in order to facilitate potential future meta-analyses for any of these individual SNPs.

To investigate whether associations of BMI, trouser/skirt size, BMI at age 20, non-occupational physical activity, height, and energy restriction during childhood and adolescence with overall and subsite-specific CRC risks in men and women were modified by the polygenic risk scores, we stratified associations by tertiles of the sex-specific polygenic risk scores and tested multiplicative interactions using the Wald test in men and women (*wald.test*, aod package in R). Participants with incomplete or inconsistent baseline questionnaires were excluded from these analyses, leaving 2191 male and 2248 female subcohort members and 2409 male and 1870 CRC cases, although the total number per analysis differed because of missing values on specific exposure variables and covariates (Fig. 1). Since BMI, trouser/skirt size, BMI at age 20, non-occupational physical activity, height, and energy restriction during childhood and adolescence have been studied as CRC risk factors after 16.3 years of follow-up in our cohort, we used the same confounder sets as before, which included established CRC risk factors and confounders derived using a backward procedure. [16, 17, 26, 39, 46–48].

Our approach of using polygenic risk scores was aimed at reducing the risk of overfitting, which can lead to inflated estimates or false positive findings. Since most of the SNPs used in this study were tagging polymorphisms that were selected to cover as much genetic variation as possible in the set of identified top-ranked genes in the mTOR-PI3K-Akt pathway, we did not have data from an independent population available on the risk-conferring allele for the majority of the SNPs. By generating a risk score in each half of the data and then merging the data back together again, we benefited from optimal power to carry out subsite-specific analyses and investigate potential effect modification by the polygenic risk scores of associations between energy balance-related factors and (subsite-specific) CRC risks in men and women separately.

All Cox models (*coxph*, survival package in R) were adjusted for the additional variance introduced by sampling the subcohort from the total cohort by entering the participant identification number as cluster term in the model (robust variance option). [35] We checked potential violations of the proportional hazards assumption by plotting the scaled Schoenfeld residuals against time and violations appeared negligible (*cox.zph*, survival package in R). Statistical significance was indicated by a *P-*value <0.05 for two-sided testing. Gene-based false discovery rate-adjusted *P*-values across men and women were calculated according to the method of Benjamini and Hochberg for *P-*values for individual SNP-CRC associations. The FDR adjustment entailed ranking *P*-values in ascending order and multiplying a predefined FDR threshold (0.20 [49]) with the inverse of the rank order over the total number of *P*-values considered to be part of the multiple testing. [50] If the original *P*-value was below 0.05 and the FDR-adjusted *P*-value, we considered the result statistically significant.

## Supplementary information

Additional file 1: **Supplemental Fig. 1.** Histogram of the sex-specific polygenic risk scores in male and female subcohort members in datasets A and B used to generate the scores.

**Additional file 2.**

## Data Availability

The authors declare that the data supporting the findings of this study are available within the article and its supplementary information files.

## References

[1] Hanahan D, Weinberg RA (2011). Hallmarks of cancer: the next generation. Cell.

[2] Zoncu R, Efeyan A, Sabatini DM (2011). mTOR: from growth signal integration to cancer, diabetes and ageing. Nat Rev Mol Cell Biol.

[3] Slattery ML, Herrick JS, Lundgreen A, Fitzpatrick FA, Curtin K, Wolff RK (2010). Genetic variation in a metabolic signaling pathway and colon and rectal cancer risk: mTOR, PTEN, STK11, RPKAA1, PRKAG2, TSC1, TSC2, PI3K and Akt1. Carcinogenesis.

[4] Campa D, Claus R, Dostal L, Stein A, Chang-Claude J, Meidtner K, Boeing H, Olsen A, Tjonneland A, Overvad K (2011). Variation in genes coding for AMP-activated protein kinase (AMPK) and breast cancer risk in the European Prospective Investigation on Cancer (EPIC). Breast Cancer Res Treat.

[5] Cao Q, Ju X, Li P, Meng X, Shao P, Cai H, Wang M, Zhang Z, Qin C, Yin C (2012). A functional variant in the MTOR promoter modulates its expression and is associated with renal cell cancer risk. PLoS One.

[6] Wang LE, Ma H, Hale KS, Yin M, Meyer LA, Liu H, Li J, Lu KH, Hennessy BT, Li X (2012). Roles of genetic variants in the PI3K and RAS/RAF pathways in susceptibility to endometrial cancer and clinical outcomes. J Cancer Res Clin Oncol.

[7] Shao J, Li Y, Zhao P, Yue X, Jiang J, Liang X, He X (2014). Association of mTOR polymorphisms with cancer risk and clinical outcomes: a meta-analysis. PLoS One.

[8] Lin L, Zhang Z, Zhang W, Wang L, Wang J (2015). Roles of genetic variants in the PI3K/PTEN pathways in susceptibility to colorectal carcinoma and clinical outcomes treated with FOLFOX regimen. Int J Clin Exp Pathol.

[9] Piao Y, Li Y, Xu Q, Liu JW, Xing CZ, Xie XD, Yuan Y (2015). Association of MTOR and AKT Gene Polymorphisms with Susceptibility and Survival of Gastric Cancer. PLoS One.

[10] Xu M, Gao Y, Yu T, Wang J, Cheng L, Cheng L, Cheng D, Zhu B (2015). Functional promoter rs2295080 T>G variant in MTOR gene is associated with risk of colorectal cancer in a Chinese population. Biomed Pharmacother.

[11] Zhao Y, Diao Y, Wang X, Lin S, Wang M, Kang H, Yang P, Dai C, Liu X, Liu K (2016). Impacts of the mTOR gene polymorphisms rs2536 and rs2295080 on breast cancer risk in the Chinese population. Oncotarget.

[12] Zining J, Lu X, Caiyun H, Yuan Y (2016). Genetic polymorphisms of mTOR and cancer risk: a systematic review and updated meta-analysis. Oncotarget.

[13] Qi GH, Wang CH, Zhang HG, Yu JG, Ding F, Song ZC, Xia QH. Comprehensive analysis of the effect of rs2295080 and rs2536 polymorphisms within the mTOR gene on cancer risk. Biosci Rep 2020, 40. 10.1042/BSR20191825 PMC7350887 32597485

[14] Slattery ML, Lundgreen A, Herrick JS, Caan BJ, Potter JD, Wolff RK (2011). Diet and colorectal cancer: analysis of a candidate pathway using SNPS, haplotypes, and multi-gene assessment. Nutr Cancer.

[15] Colorectal cancer | Continuous Update Project | WCRF [Internet]. [cited February 21, 2020]. Available at: http://www.wcrf.org/cancer_research/cup/key_findings/colorectal_cancer.php.

[16] Hughes LA, Simons CC, van den Brandt PA, Goldbohm RA, van Engeland M, Weijenberg MP (2011). Body size and colorectal cancer risk after 16.3 years of follow-up: an analysis from the Netherlands Cohort Study. Am J Epidemiol.

[17] Simons CC, Hughes LA, van Engeland M, Goldbohm RA, van den Brandt PA, Weijenberg MP (2013). Physical activity, occupational sitting time, and colorectal cancer risk in the Netherlands cohort study. Am J Epidemiol.

[18] Johnson SM, Gulhati P, Rampy BA, Han Y, Rychahou PG, Doan HQ, Weiss HL, Evers BM (2010). Novel expression patterns of PI3K/Akt/mTOR signaling pathway components in colorectal cancer. J Am Coll Surg.

[19] Kuramochi H, Nakamura A, Nakajima G, Kaneko Y, Araida T, Yamamoto M, Hayashi K (2016). PTEN mRNA expression is less pronounced in left- than right-sided colon cancer: a retrospective observational study. BMC Cancer.

[20] Day FL, Jorissen RN, Lipton L, Mouradov D, Sakthianandeswaren A, Christie M, Li S, Tsui C, Tie J, Desai J (2013). PIK3CA and PTEN gene and exon mutation-specific clinicopathologic and molecular associations in colorectal cancer. Clin Cancer Res.

[21] Lin J, Wang J, Greisinger AJ, Grossman HB, Forman MR, Dinney CP, Hawk ET, Wu X (2010). Energy balance, the PI3K-AKT-mTOR pathway genes, and the risk of bladder cancer. Cancer Prev Res (Phila).

[22] Shu X, Lin J, Wood CG, Tannir NM, Wu X (2013). Energy balance, polymorphisms in the mTOR pathway, and renal cell carcinoma risk. J Natl Cancer Inst.

[23] Zhu J, Wang M, Zhu M, He J, Wang JC, Jin L, Wang XF, Xiang JQ, Wei Q (2015). Associations of PI3KR1 and mTOR polymorphisms with esophageal squamous cell carcinoma risk and gene-environment interactions in Eastern Chinese populations. Sci Rep.

[24] Cheng TY, Shankar J, Zirpoli G, Roberts MR, Hong CC, Bandera EV, Ambrosone CB, Yao S (2016). Genetic variants in the mTOR pathway and interaction with body size and weight gain on breast cancer risk in African-American and European American women. Cancer Causes Control.

[25] Moore T, Beltran L, Carbajal S, Strom S, Traag J, Hursting SD, DiGiovanni J (2008). Dietary energy balance modulates signaling through the Akt/mammalian target of rapamycin pathways in multiple epithelial tissues. Cancer Prev Res (Phila).

[26] Simons CC, Schouten LJ, Godschalk R, van Engeland M, van den Brandt PA, van Schooten FJ, Weijenberg MP (2015). Body size, physical activity, genetic variants in the insulin-like growth factor pathway and colorectal cancer risk. Carcinogenesis.

[27] Kim DH, Sarbassov DD, Ali SM, King JE, Latek RR, Erdjument-Bromage H, Tempst P, Sabatini DM (2002). mTOR interacts with raptor to form a nutrient-sensitive complex that signals to the cell growth machinery. Cell.

[28] Goldbohm RA, van ’t Veer P, van den Brandt PA, van ’t Hof MA, Brants HA, Sturmans F, Hermus RJ (1995). Reproducibility of a food frequency questionnaire and stability of dietary habits determined from five annually repeated measurements. Eur J Clin Nutr.

[29] van den Brandt PA, Goldbohm RA, van ’t Veer P, Volovics A, Hermus RJ, Sturmans F (1990). A large-scale prospective cohort study on diet and cancer in The Netherlands. J Clin Epidemiol.

[30] Goldbohm RA, van den Brandt PA, Brants HA, van’t Veer P, Al M, Sturmans F, Hermus RJ (1994). Validation of a dietary questionnaire used in a large-scale prospective cohort study on diet and cancer. Eur J Clin Nutr.

[31] van Breda SG, Hogervorst JG, Schouten LJ, Knaapen AM, van Delft JH, Goldbohm RA, van Schooten FJ, van den Brandt PA (2007). Toenails: an easily accessible and long-term stable source of DNA for genetic analyses in large-scale epidemiological studies. Clin Chem.

[32] Hogervorst JG, Godschalk RW, van den Brandt PA, Weijenberg MP, Verhage BA, Jonkers L, Goessens J, Simons CC, Vermeesch JR, van Schooten FJ, Schouten LJ (2014). DNA from nails for genetic analyses in large-scale epidemiologic studies. Cancer Epidemiol Biomarkers Prev.

[33] Van den Brandt PA, Schouten LJ, Goldbohm RA, Dorant E, Hunen PM (1990). Development of a record linkage protocol for use in the Dutch Cancer Registry for Epidemiological Research. Int J Epidemiol.

[34] Goldbohm RA, van den Brandt PA, Dorant E (1994). Estimation of the coverage of Dutch municipalities by cancer registries and PALGA based on hospital discharge data. Tijdschr Soc Gezondheidsz.

[35] Barlow WE (1994). Robust variance estimation for the case-cohort design. Biometrics.

[36] Skeie G, Mode N, Henningsen M, Borch KB (2015). Validity of self-reported body mass index among middle-aged participants in the Norwegian Women and Cancer study. Clin Epidemiol.

[37] Wright FL, Green J, Reeves G, Beral V, Cairns BJ (2015). Million Women Study c: Validity over time of self-reported anthropometric variables during follow-up of a large cohort of UK women. BMC Med Res Methodol.

[38] Hughes LA, Schouten LJ, Goldbohm RA, van den Brandt PA, Weijenberg MP (2009). Self-reported clothing size as a proxy measure for body size. Epidemiology.

[39] Simons CC, Schouten LJ, Godschalk RW, van Engeland M, van den Brandt PA, van Schooten FJ, Weijenberg MP (2017). Energy restriction at young age, genetic variants in the insulin-like growth factor pathway and colorectal cancer risk in the Netherlands Cohort Study. Int J Cancer.

[40] de Bakker PI, Yelensky R, Pe’er I, Gabriel SB, Daly MJ, Altshuler D (2005). Efficiency and power in genetic association studies. Nat Genet.

[41] Berndt SI, Gustafsson S, Magi R, Ganna A, Wheeler E, Feitosa MF, Justice AE, Monda KL, Croteau-Chonka DC, Day FR (2013). Genome-wide meta-analysis identifies 11 new loci for anthropometric traits and provides insights into genetic architecture. Nat Genet.

[42] Comuzzie AG, Cole SA, Laston SL, Voruganti VS, Haack K, Gibbs RA, Butte NF (2012). Novel genetic loci identified for the pathophysiology of childhood obesity in the Hispanic population. PLoS One.

[43] Lango Allen H, Estrada K, Lettre G, Berndt SI, Weedon MN, Rivadeneira F, Willer CJ, Jackson AU, Vedantam S, Raychaudhuri S (2010). Hundreds of variants clustered in genomic loci and biological pathways affect human height. Nature.

[44] He M, Xu M, Zhang B, Liang J, Chen P, Lee JY, Johnson TA, Li H, Yang X, Dai J (2015). Meta-analysis of genome-wide association studies of adult height in East Asians identifies 17 novel loci. Hum Mol Genet.

[45] Wood AR, Esko T, Yang J, Vedantam S, Pers TH, Gustafsson S, Chu AY, Estrada K, Luan J, Kutalik Z (2014). Defining the role of common variation in the genomic and biological architecture of adult human height. Nat Genet.

[46] Hughes LA, van den Brandt PA, de Bruine AP, Wouters KA, Hulsmans S, Spiertz A, Goldbohm RA, de Goeij AF, Herman JG, Weijenberg MP, van Engeland M (2009). Early life exposure to famine and colorectal cancer risk: a role for epigenetic mechanisms. PLoS One.

[47] Hughes LA, van den Brandt PA, Goldbohm RA, de Goeij AF, de Bruine AP, van Engeland M, Weijenberg MP (2010). Childhood and adolescent energy restriction and subsequent colorectal cancer risk: results from the Netherlands Cohort Study. Int J Epidemiol.

[48] Simons CC, van den Brandt PA, Stehouwer CD, van Engeland M, Weijenberg MP (2014). Body size, physical activity, early-life energy restriction, and associations with methylated insulin-like growth factor-binding protein genes in colorectal cancer. Cancer Epidemiol Biomarkers Prev.

[49] Smith NL, Hindorff LA, Heckbert SR, Lemaitre RN, Marciante KD, Rice K, Lumley T, Bis JC, Wiggins KL, Rosendaal FR, Psaty BM (2007). Association of genetic variations with nonfatal venous thrombosis in postmenopausal women. JAMA.

[50] Benjamini Y, Drai D, Elmer G, Kafkafi N, Golani I (2001). Controlling the false discovery rate in behavior genetics research. Behav Brain Res.

