# Supplementary figures and images for: Polymorphisms in the mTOR-PI3K-Akt pathway, energy balance-related exposures and colorectal cancer risk in the Netherlands Cohort Study
Source: BioData Min. 2022 Jan 10;15:2. doi: 10.1186/s13040-021-00286-3 (PMC8751328; doi:10.1186/s13040-021-00286-3)

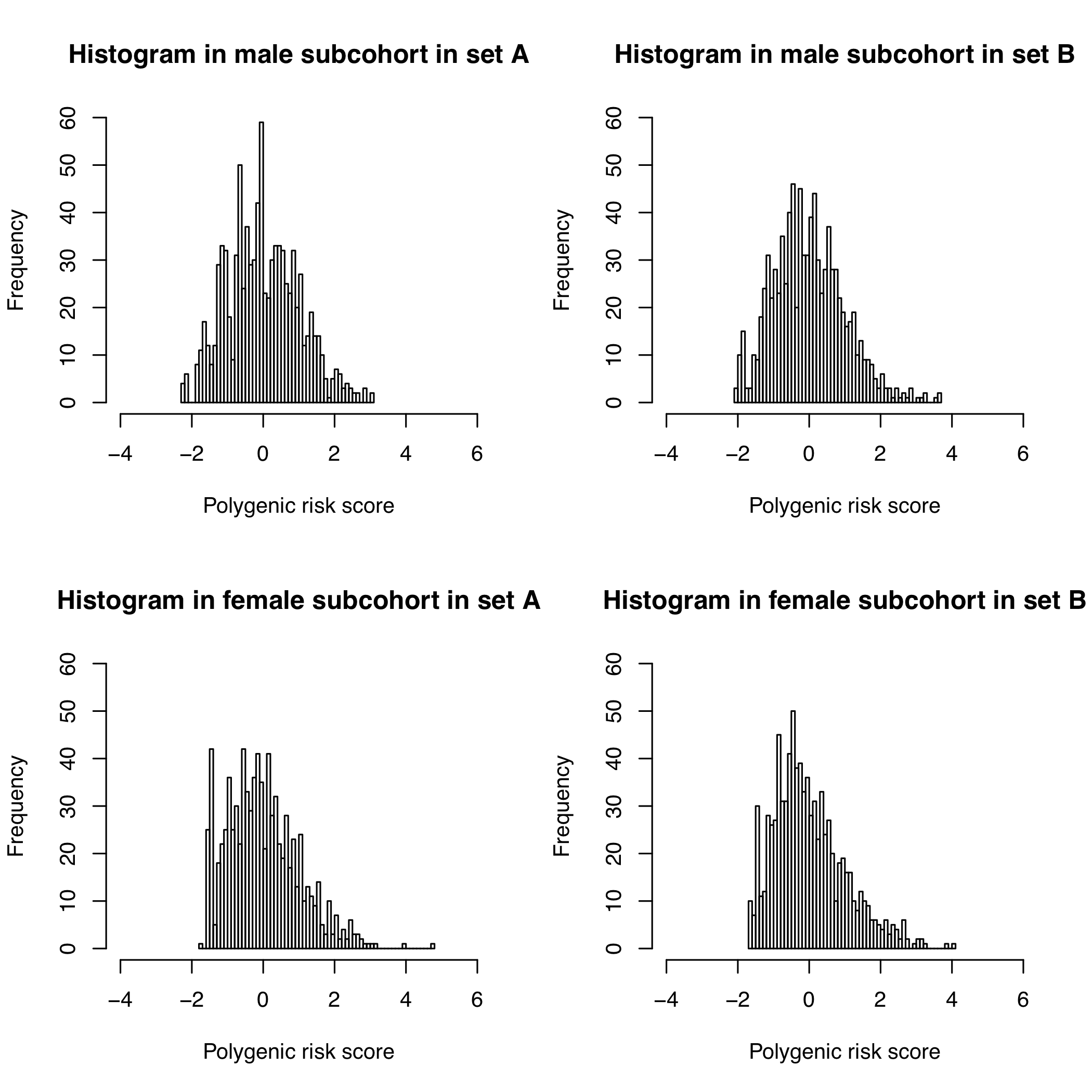

Supplement: Supplementary file 1 — Supplemental Fig. 1. Histogram of the sex-specific polygenic risk scores in male and female subcohort members in datasets A and B used to generate the scores. [file 13040_2021_286_MOESM1_ESM.tif]
