# Supplementary material for: Polymorphisms in the mTOR-PI3K-Akt pathway, energy balance-related exposures and colorectal cancer risk in the Netherlands Cohort Study
Source: BioData Min. 2022 Jan 10;15:2. doi: 10.1186/s13040-021-00286-3 (PMC8751328; doi:10.1186/s13040-021-00286-3)
Supplement: Supplementary file 2 — Additional file 2. [file 13040_2021_286_MOESM2_ESM.docx]

| **Supplemental Table 1.** Top-ranked genes in the mTOR-PI3k-Akt pathway^a^ | | |
| --- | --- | --- |
| **Rank** | **Gene** | **Abbreviation explained** |
| 1 | *MTOR (alias FRAP1) ^b^* | Mechanistic Target Of Rapamycin |
| 2 | *TSC2 ^b^* | Tuberous Sclerosis 2 |
| 3 | *PDPK1 (alias PDK1) ^b^* | 3-Phosphoinositide Dependent Protein Kinase 1 |
| 4 | *EIF4EBP1 (alias 4EBP1) ^b^* | Eukaryotic Translation Initiation Factor 4E Binding Protein 1 |
| 5 | *IRS1* | Insulin Receptor Substrate 1 |
| 6 | *RPS6KB1 (alias S6K1)* | Ribosomal Protein S6 Kinase B1 |
| 7 | *RPS6KB2 (alias S6K2) ^b^* | Ribosomal Protein S6 Kinase B2 |
| 8 | *AKT3 ^b^* | AKT Serine/Threonine Kinase 3 |
| 9 | *AKT1* | AKT Serine/Threonine Kinase 1 |
| 10 | *AKT2 ^b^* | AKT Serine/Threonine Kinase 2 |
| *^c^* | *IGF1R* | Insulin Like Growth Factor 1 Receptor |
| *^c^* | *INSR* | Insulin Receptor |
| *^c^* | *RICTOR* | RPTOR Independent Companion Of MTOR Complex 2 |
| *^c^* | *RPTOR* | Regulatory Associated Protein Of MTOR Complex 1 |
| ^a^ These genes were identified based on their relative betweenness centrality, which provides an indication of the strength of node involvement in the information flow through a network using the Kyoto Ecyclopedia of Genes and Genomes (KEGG) mTOR signaling (map04150) as input (<http://www.genome.jp/kegg/>) (R software, version 3.2.2, KeggGraph package).  *^b^* Tagging variants for these 7 genes could be included in the final genotyping assay (not all variants could be included together because of sequence incompatibilities between the sequences flanking the SNPs).  *^c^* In order to make optimal use of the genotyping assay, the assay was filled up as much as possible with single genome-wide association study (GWAS) hits for anthropometric traits, physical activity, or CRC annotated to mTOR-PI3K-Akt pathway genes (<https://www.ebi.ac.uk/gwas/>), which led these genes to also be represented in the genotyping assay. | | |

| **Supplemental Table 2.** Single nucleotide polymorphisms^a^ in the mTOR-PI3k-Akt pathway genotyped within the Netherlands Cohort Study in subcohort members and CRC cases (20.3 years follow-up) | | | | | | | | | |
| --- | --- | --- | --- | --- | --- | --- | --- | --- | --- |
| **Variant** | **Gene Annotation** | **Locus** | **Major Allele** | **Minor Allele** | **Minor Allele**  **Frequency^a^** | **N (%) Homozygotes**  **Common Allele^b^** | **N (%)**  **Heterozygotes^b^** | **N (%) Homozygotes**  **Minor Allele^b^** | ***P* for Hardy-Weinberg**  **Equilibrium^b^** |
| rs2295080 ^c^ | *MTOR* (from literature) | 1:11322628 | T | G | 0.31 | 1768 (47.9) | 1562 (42.3) | 361 (9.8) | 0.56 |
| rs1057079 ^d^ | *MTOR* | 1:11205058 | T | C | 0.27 | 1990 (54.0) | 1429 (38.8) | 265 (7.2) | 0.70 |
| rs12918803 ^c^ | *TSC2* | 16:2142353 | G | A | 0.08 | 3183 (85.6) | 505 (13.6) | 30 (0.8) | 0.05 |
| rs1800720 ^d^ | *TSC2* | 16:2105400 | C | T | 0.09 | 3047 (82.5) | 608 (16.5) | 37 (1.0) | 0.28 |
| rs2074969 ^e^ | *TSC2* | 16:2111230 | A | G | 0.50 | 882 (24.1) | 1861 (50.9) | 917 (25.1) | 0.30 |
| rs2516739 ^d^ | *TSC2* | 16:2097158 | G | A | 0.21 | 2311 (62.1) | 1245 (33.5) | 164 (4.4) | 0.82 |
| rs9928737 ^e^ | *TSC2* | 16:2132731 | G | A | 0.17 | 2584 (69.9) | 997 (27.0) | 118 (3.2) | 0.07 |
| rs6723872 ^c, d^ | *PDK1* | 2:173453018 | A | G | 0.17 | 2575 (69.8) | 1013 (27.5) | 103 (2.8) | 0.78 |
| rs6605631 ^d^ | *EIF4EBP1* | 8:37902453 | T | C | 0.23 | 2179 (59.4) | 1298 (35.4) | 194 (5.3) | 0.97 |
| rs12787021 ^c, d^ | *RPS6KB2* | 11:67204342 | A | G | 0.45 | 1122 (30.2) | 1874 (50.5) | 716 (19.3) | 0.18 |
| rs1352162 ^c^ | *AKT3* | 1:243717682 | G | A | 0.31 | 1749 (47.8) | 1556 (42.5) | 353 (9.7) | 0.80 |
| rs14403 ^c, d^ | *AKT3* | 1:243663893 | C | T | 0.25 | 2132 (57.0) | 1341 (35.8) | 270 (7.2) | 0.003 |
| rs3006939 ^d^ | *AKT3* | 1:243677834 | G | A | 0.23 | 2201 (60.2) | 1254 (34.3) | 202 (5.5) | 0.18 |
| rs7523198 ^c^ | *AKT3* | 1:243702266 | T | C | 0.11 | 2919 (78.8) | 724 (19.5) | 63 (1.7) | 0.02 |
| rs7523742 ^c^ | *AKT3* | 1:243688150 | G | A | 0.07 | 3187 (86.5) | 470 (12.8) | 26 (0.7) | 0.06 |
| rs946824 ^d^ | *AKT3* | 1:243684019 | C | T | 0.11 | 2927 (79.2) | 708 (19.2) | 59 (1.6) | 0.03 |
| rs16974157 ^c, d^ | *AKT2* | 19:40772924 | C | A | 0.15 | 2645 (71.8) | 960 (26.1) | 81 (2.2) | 0.58 |
| rs7250897 ^e^ | *AKT2* | 19:40783233 | C | T | 0.30 | 1814 (49.6) | 1510 (41.3) | 335 (9.2) | 0.42 |
| rs874269 ^c, d^ | *AKT2* | 19:40731945 | C | T | 0.08 | 3149 (84.9) | 534 (14.4) | 26 (0.7) | 0.52 |
| rs2871865 | *IGF1R* (from GWAS) | 15:99194896 | C | G | 0.10 | 3002 (80.7) | 666 (17.9) | 52 (1.4) | 0.03 |
| rs891088 ^c, d^ | *INSR* (from GWAS) | 19:7184762 | A | G | 0.26 | 2000 (55.1) | 1396 (38.4) | 236 (6.5) | 0.72 |
| rs2043112 ^e^ | *RICTOR* (from GWAS) | 5:38955796 | G | A | 0.42 | 1230 (33.3) | 1812 (49.0) | 653 (17.7) | 0.75 |
| rs7503807 ^e^ | *RPTOR* (from GWAS) | 17:78591111 | A | C | 0.45 | 1163 (31.2) | 1794 (48.1) | 771 (20.7) | 0.10 |
| rs1051424 ^e^ | *S6K1* (from GWAS) | 17:58024324 | A | G | 0.06 | 3173 (90.7) | 199 (5.7) | 125 (3.6) | <0.001 |
| Abbreviations: CRC, colorectal cancer  ^a^ Tagging single nucleotide polymorphisms, unless otherwise specified.  ^b^ Based on the subcohort in the Netherlands Cohort Study, which was selected at random and independent of any exposure at baseline in 1986, and thus represents the whole cohort.  ^c^ Included in the polygenic risk score for women.  ^d^ Included in the polygenic risk score for men.  ^e^ Not included in any polygenic risk score because of inconsistent betas (see Supplemental Table 3). | | | | | | | | | |

| **Supplemental Table 3.** Regression coefficients and corresponding standard errors for associations between individual SNPs in the mTOR-PI3k-Akt pathway and colorectal cancer risk in datasets A and B in men and women from the Netherlands Cohort Study after 20.3 years follow-up; datasets A and B both comprised half of the data and were used to calculate standard error weighted regression coefficients used to generate polygenic risk scores (see note) | | | | | | | | | | | | | | | |
| --- | --- | --- | --- | --- | --- | --- | --- | --- | --- | --- | --- | --- | --- | --- | --- |
|  |  | **Men** | | | | | | | **Women** | | | | | | |
|  |  | **Dataset A** | | | **Dataset B** | | | **Incon-**  **sistent**  **betas?**  **(1, yes;**  **0, no)** | **Dataset A** | | | **Dataset B** | | | **Incon-**  **sistent**  **betas?**  **(1, yes;**  **0, no)** |
| **Variant** | **Gene Annotation** | **Beta^a^** | **SE** | **(Absolute Beta) / SE^a^** | **Beta** | **SE** | **(Absolute Beta) / SE^a^** |  | **Beta** | **SE** | **(Absolute Beta) / SE^a^** | **Beta** | **SE** | **(Absolute Beta) / SE^a^** |  |
| rs1057079 | *MTOR* | 0.08 | 0.05 | 1.55 | 0.07 | 0.05 | 1.35 | 0 ^b^ | -0.24 | 0.06 | 3.89 | 0.02 | 0.06 | 0.27 | 1 |
| rs2295080 | *MTOR* (from literature) | 0.10 | 0.05 | 2.11 | 0.08 | 0.05 | 1.64 | 0 | -0.17 | 0.06 | 2.84 | -0.01 | 0.06 | 0.14 | 0 |
| rs12918803 | *TSC2* | -0.08 | 0.08 | 0.94 | 0.13 | 0.08 | 1.54 | 1 | 0.07 | 0.10 | 0.70 | 0.10 | 0.10 | 0.97 | 0 |
| rs1800720 | *TSC2* | 0.08 | 0.08 | 1.11 | 0.08 | 0.08 | 1.01 | 0 | 0.11 | 0.09 | 1.26 | -0.03 | 0.09 | 0.35 | 1 |
| rs2074969 | *TSC2* | -0.11 | 0.05 | 2.39 | 0.01 | 0.05 | 0.12 | 1 | -0.10 | 0.05 | 1.86 | 0.05 | 0.05 | 0.91 | 1 |
| rs2516739 | *TSC2* | 0.13 | 0.06 | 2.27 | 0.04 | 0.06 | 0.71 | 0 | -0.01 | 0.06 | 0.13 | 0.001 | 0.06 | 0.02 | 1 |
| rs9928737 | *TSC2* | -0.01 | 0.06 | 0.16 | 0.02 | 0.06 | 0.35 | 1 | 0.05 | 0.07 | 0.69 | -0.04 | 0.07 | 0.52 | 1 |
| rs6723872 | *PDK1* | 0.01 | 0.06 | 0.12 | 0.08 | 0.06 | 1.36 | 0 | 0.002 | 0.07 | 0.03 | 0.08 | 0.07 | 1.19 | 0 |
| rs6605631 | *EIF4EBP1* | -0.07 | 0.05 | 1.21 | -0.02 | 0.06 | 0.35 | 0 | 0.05 | 0.06 | 0.84 | -0.15 | 0.07 | 2.22 | 1 |
| rs12787021 | *AKT3* | 0.09 | 0.05 | 2.00 | 0.08 | 0.05 | 1.78 | 0 | -0.05 | 0.05 | 0.89 | -0.01 | 0.05 | 0.12 | 0 |
| rs1352162 | *AKT3* | -0.01 | 0.05 | 0.23 | 0.18 | 0.05 | 3.39 | 1 | 0.20 | 0.06 | 3.59 | 0.03 | 0.06 | 0.43 | 0 |
| rs14403 | *AKT3* | 0.06 | 0.05 | 1.22 | 0.09 | 0.05 | 1.59 | 0 | -0.02 | 0.06 | 0.29 | -0.05 | 0.06 | 0.86 | 0 |
| rs3006939 | *AKT3* | 0.02 | 0.05 | 0.28 | 0.08 | 0.06 | 1.45 | 0 | 0.07 | 0.06 | 1.15 | -0.11 | 0.07 | 1.60 | 1 |
| rs7523198 | *AKT3* | -0.01 | 0.07 | 0.18 | 0.19 | 0.07 | 2.74 | 1 | 0.28 | 0.08 | 3.74 | 0.13 | 0.08 | 1.64 | 0 |
| rs7523742 | *AKT3* | -0.01 | 0.09 | 0.08 | 0.10 | 0.09 | 1.14 | 1 | 0.22 | 0.10 | 2.30 | 0.21 | 0.10 | 2.08 | 0 |
| rs946824 | *AKT3* | 0.02 | 0.07 | 0.31 | 0.21 | 0.07 | 3.00 | 0 | 0.32 | 0.08 | 4.16 | 0.16 | 0.08 | 1.92 | 0 ^c^ |
| rs16974157 | *AKT2* | -0.04 | 0.07 | 0.59 | -0.03 | 0.06 | 0.42 | 0 | -0.11 | 0.08 | 1.35 | -0.16 | 0.08 | 2.09 | 0 |
| rs7250897 | *AKT2* | 0.08 | 0.05 | 1.66 | -0.06 | 0.05 | 1.16 | 1 | -0.06 | 0.06 | 1.00 | 0.08 | 0.06 | 1.45 | 1 |
| rs874269 | *AKT2* | -0.04 | 0.09 | 0.50 | -0.12 | 0.09 | 1.31 | 0 | 0.17 | 0.09 | 1.80 | 0.07 | 0.10 | 0.69 | 0 |
| rs2871865 | *IGF1R* (from GWAS) | 0.06 | 0.08 | 0.72 | -0.12 | 0.08 | 1.54 | 1 | 0.08 | 0.08 | 0.92 | -0.12 | 0.09 | 1.35 | 1 |
| rs891088 | *INSR* (from GWAS) | -0.02 | 0.05 | 0.43 | -0.07 | 0.05 | 1.25 | 0 | 0.14 | 0.06 | 2.27 | 0.11 | 0.06 | 1.78 | 0 |
| rs2043112 | *RICTOR* (from GWAS) | -0.09 | 0.05 | 1.91 | 0.01 | 0.05 | 0.27 | 1 | 0.07 | 0.05 | 1.38 | -0.04 | 0.05 | 0.74 | 1 |
| rs7503807 | *RPTOR* (from GWAS) | 0.03 | 0.05 | 0.63 | -0.01 | 0.04 | 0.24 | 1 | -0.01 | 0.05 | 0.17 | 0.06 | 0.05 | 1.10 | 1 |
| rs1051424 | *S6K1* (from GWAS) | 0.02 | 0.07 | 0.29 | -0.16 | 0.08 | 1.98 | 1 | 0.22 | 0.09 | 2.50 | -0.10 | 0.10 | 1.06 | 1 |
| Note: The polygenic risk scores were calculated based on SNPs showing the same direction of effect in both dataset A and dataset B. Scores between men and women were allowed to include different SNPs. The scores were calculated by multiplying the number of risk alleles in one set with the standard error weighted regression coefficient from the other set and aggregating the resulting values across SNPs into a single score for each individual. In case of a negative regression coefficient, the SNP coding was reversed from ‘0’ for homozygotes for the major allele, ‘1’ for heterozygotes, and ‘2’ for homozygotes for the minor allele to ‘0’ for heterozygotes for the minor allele, ‘1’ for heterozygotes, and ‘2’ for homozygotes for the major allele.  ^a^ This table shows rounded values, but calculations were performed without rounding off values, which is why the standard error weighted regression coefficients shown in this table deviate slightly from the values that can be calculated by hand using the rounded values.  ^b^ Not included in the polygenic risk score because this SNP was in LD with SNP rs2295080 at r^2^>0.6.  ^c^ Not included in the polygenic risk score because this SNP was in LD with SNP rs7523742 at r^2^>0.6. | | | | | | | | | | | | | | | |

| **Supplemental Table 4.** Individual polymorphisms in the mTOR-PI3K-Akt pathway in relation to colorectal cancer risk in men and women from the Netherlands Cohort Study (20.3 years of follow-up) | | | | | | | | | | | | | | |
| --- | --- | --- | --- | --- | --- | --- | --- | --- | --- | --- | --- | --- | --- | --- |
|  |  |  | **Men** | | | | | | **Women** | | | | | |
| **Gene** | **Variant** | **Comparison** | **PT at risk** | **N cases** | **HR^a^** | **(95% CI)** | ***P*** **^b^** |  | **PT at risk** | **N cases** | **HR^a^** | **(95% CI)** | ***P* ^b^** |  |
| MTOR | rs1057079 | TC vs. TT | 11040/15844 | 753/992 | 1.10 | (0.96,1.27) | 0.18 |  | 12053/16083 | 518/797 | 0.87 | (0.75,1.01) | 0.07 |  |
|  |  | CC vs. TT | 1937/15844 | 136/992 | 1.12 | (0.85,1.46) | 0.42 |  | 2261/16083 | 94/797 | 0.86 | (0.64,1.15) | 0.30 |  |
|  |  | Per minor allele | 28821 | 1881 | 1.08 | (0.97,1.20) | 0.17 |  | 30397 | 1409 | 0.90 | (0.80,1.01) | 0.07 |  |
|  | rs2295080 | TG vs. TT | 11826/14270 | 827/870 | 1.17 | (1.01,1.35) | 0.03 |  | 13222/14029 | 587/694 | 0.91 | (0.78,1.05) | 0.20 |  |
|  | (from literature) | GG vs. TT | 2745/14270 | 185/870 | 1.12 | (0.88,1.41) | 0.36 |  | 3085/14029 | 129/694 | 0.87 | (0.67,1.12) | 0.27 |  |
|  |  | Per minor allele | 28841 | 1882 | 1.09 | (0.99,1.21) | 0.08 |  | 30337 | 1410 | 0.92 | (0.82,1.03) | 0.15 |  |
| TSC2 | rs12918803 | GA vs. GG | 3910/24629 | 279/1591 | 1.10 | (0.91,1.34) | 0.32 |  | 4183/26018 | 203/1198 | 1.08 | (0.88,1.32) | 0.48 |  |
|  |  | AA vs. GG | 302/24629 | 12/1591 | 0.57 | (0.27,1.20) | 0.14 |  | 176/26018 | 10/1198 | 1.30 | (0.52,3.22) | 0.58 |  |
|  |  | Per minor allele | 28841 | 1882 | 1.02 | (0.86,1.20) | 0.83 |  | 30377 | 1411 | 1.09 | (0.90,1.31) | 0.39 |  |
|  | rs1800720 | CT vs. CC | 4755/23803 | 339/1528 | 1.12 | (0.94,1.34) | 0.20 |  | 5247/24865 | 240/1154 | 1.01 | (0.84,1.23) | 0.90 |  |
|  |  | TT vs. CC | 283/23803 | 15/1528 | 0.86 | (0.41,1.78) | 0.68 |  | 285/24865 | 16/1154 | 1.30 | (0.64,2.61) | 0.47 |  |
|  |  | Per minor allele | 28841 | 1882 | 1.08 | (0.92,1.27) | 0.33 |  | 30397 | 1410 | 1.04 | (0.87,1.23) | 0.67 |  |
|  | rs2074969 | AG vs. AA | 14673/6970 | 936/488 | 0.90 | (0.76,1.06) | 0.21 |  | 15159/7716 | 701/367 | 0.95 | (0.80,1.14) | 0.60 |  |
|  |  | GG vs. AA | 7186/6970 | 457/488 | 0.90 | (0.74,1.09) | 0.27 |  | 7502/7716 | 341/367 | 0.95 | (0.78,1.17) | 0.64 |  |
|  |  | Per minor allele | 28829 | 1881 | 0.95 | (0.86,1.04) | 0.27 |  | 30377 | 1409 | 0.98 | (0.88,1.08) | 0.64 |  |
|  | rs2516739 | GA vs. GG | 10074/17767 | 664/1131 | 1.05 | (0.91,1.21) | 0.52 |  | 9818/18907 | 472/873 | 1.05 | (0.90,1.23) | 0.51 |  |
|  |  | AA vs. GG | 1000/17767 | 87/1131 | 1.32 | (0.92,1.89) | 0.13 |  | 1673/18907 | 65/873 | 0.87 | (0.62,1.21) | 0.41 |  |
|  |  | Per minor allele | 28841 | 1882 | 1.09 | (0.96,1.23) | 0.17 |  | 30397 | 1410 | 1.00 | (0.88,1.12) | 0.95 |  |
|  | rs9928737 | GA vs. GG | 8129/19922 | 541/1291 | 1.03 | (0.89,1.20) | 0.69 |  | 8012/21211 | 404/970 | 1.12 | (0.95,1.32) | 0.16 |  |
|  |  | AA vs. GG | 790/19922 | 49/1291 | 0.91 | (0.60,1.39) | 0.66 |  | 1174/21211 | 36/970 | 0.70 | (0.46,1.06) | 0.09 |  |
|  |  | Per minor allele | 28841 | 1881 | 1.01 | (0.89,1.14) | 0.93 |  | 30397 | 1410 | 1.01 | (0.89,1.15) | 0.91 |  |
| PDK1 | rs6723872 | AG vs. AA | 8022/20090 | 532/1295 | 1.03 | (0.89,1.20) | 0.66 |  | 8151/21249 | 396/973 | 1.06 | (0.90,1.24) | 0.50 |  |
|  |  | GG vs. AA | 682/20090 | 51/1295 | 1.16 | (0.75,1.79) | 0.50 |  | 887/21249 | 42/973 | 1.04 | (0.68,1.60) | 0.86 |  |
|  |  | Per minor allele | 28794 | 1878 | 1.05 | (0.92,1.19) | 0.48 |  | 30286 | 1411 | 1.04 | (0.91,1.19) | 0.53 |  |
| EIF4EBP1 | rs6605631 | TC vs. TT | 9860/17356 | 622/1152 | 0.93 | (0.81,1.08) | 0.36 |  | 10828/17953 | 475/860 | 0.91 | (0.78,1.06) | 0.24 |  |
|  |  | CC vs. TT | 1625/17356 | 106/1152 | 0.97 | (0.72,1.30) | 0.82 |  | 1617/17953 | 76/860 | 1.02 | (0.74,1.41) | 0.91 |  |
|  |  | Per minor allele | 28841 | 1880 | 0.96 | (0.85,1.07) | 0.45 |  | 30397 | 1411 | 0.95 | (0.84,1.08) | 0.46 |  |
| RPS6KB2 | rs12787021 | AG vs. AA | 14416/8863 | 909/555 | 1.01 | (0.87,1.18) | 0.89 |  | 15522/9072 | 708/437 | 0.95 | (0.80,1.12) | 0.52 |  |
|  |  | GG vs. AA | 5563/8863 | 417/555 | 1.20 | (0.99,1.46) | 0.06 |  | 5783/9072 | 265/437 | 0.95 | (0.77,1.17) | 0.63 |  |
|  |  | Per minor allele | 28841 | 1881 | 1.09 | (0.99,1.20) | 0.08 |  | 30377 | 1410 | 0.97 | (0.88,1.08) | 0.59 |  |
| AKT3 | rs1352162 | GA vs. GG | 12594/13469 | 794/870 | 0.99 | (0.86,1.14) | 0.88 |  | 12617/14933 | 625/642 | 1.16 | (1.00,1.35) | 0.06 |  |
|  |  | AA vs. GG | 2722/13469 | 214/870 | 1.25 | (0.99,1.58) | 0.06 |  | 2716/14933 | 142/642 | 1.23 | (0.96,1.59) | 0.10 |  |
|  |  | Per minor allele | 28786 | 1878 | 1.07 | (0.97,1.19) | 0.19 |  | 30266 | 1409 | 1.13 | (1.01,1.26) | 0.03 |  |
|  | rs14403 | CT vs. CC | 10357/16860 | 675/1074 | 1.02 | (0.88,1.17) | 0.80 |  | 10967/17533 | 534/810 | 1.06 | (0.91,1.23) | 0.46 |  |
|  |  | TT vs. CC | 1624/16860 | 133/1074 | 1.28 | (0.97,1.69) | 0.09 |  | 1885/17533 | 67/810 | 0.76 | (0.55,1.06) | 0.10 |  |
|  |  | Per minor allele | 28841 | 1882 | 1.08 | (0.96,1.20) | 0.20 |  | 30385 | 1411 | 0.97 | (0.86,1.09) | 0.61 |  |
|  | rs3006939 | GA vs. GG | 10222/17094 | 663/1103 | 1.00 | (0.87,1.16) | 0.96 |  | 10217/18521 | 497/851 | 1.07 | (0.92,1.25) | 0.37 |  |
|  |  | AA vs. GG | 1525/17094 | 116/1103 | 1.19 | (0.89,1.60) | 0.23 |  | 1659/18521 | 63/851 | 0.82 | (0.59,1.15) | 0.25 |  |
|  |  | Per minor allele | 28841 | 1882 | 1.05 | (0.93,1.17) | 0.43 |  | 30397 | 1411 | 0.99 | (0.88,1.12) | 0.93 |  |
|  | rs7523198 | TC vs. TT | 5483/22774 | 407/1448 | 1.19 | (1.00,1.40) | 0.05 |  | 6147/23835 | 316/1061 | 1.16 | (0.97,1.38) | 0.11 |  |
|  |  | CC vs. TT | 584/22774 | 27/1448 | 0.75 | (0.45,1.26) | 0.28 |  | 415/23835 | 34/1061 | 2.03 | (1.18,3.50) | 0.01 |  |
|  |  | Per minor allele | 28841 | 1882 | 1.08 | (0.94,1.25) | 0.26 |  | 30397 | 1411 | 1.23 | (1.05,1.44) | 0.009 |  |
|  | rs7523742 | GA vs. GG | 3454/25057 | 235/1627 | 1.08 | (0.88,1.33) | 0.46 |  | 4042/26066 | 207/1183 | 1.15 | (0.94,1.41) | 0.19 |  |
|  |  | AA vs. GG | 187/25057 | 10/1627 | 0.80 | (0.34,1.88) | 0.62 |  | 115/26066 | 14/1183 | 3.19 | (1.28,7.95) | 0.01 |  |
|  |  | Per minor allele | 28698 | 1872 | 1.04 | (0.87,1.26) | 0.64 |  | 30222 | 1404 | 1.24 | (1.02,1.50) | 0.03 |  |
|  | rs946824 | CT vs. CC | 5391/22916 | 401/1453 | 1.20 | (1.01,1.42) | 0.04 |  | 6024/24010 | 313/1064 | 1.18 | (0.99,1.40) | 0.07 |  |
|  |  | TT vs. CC | 534/22916 | 28/1453 | 0.86 | (0.51,1.44) | 0.57 |  | 364/24010 | 34/1064 | 2.33 | (1.32,4.10) | 0.003 |  |
|  |  | Per minor allele | 28841 | 1882 | 1.11 | (0.97,1.28) | 0.14 |  | 30397 | 1411 | 1.27 | (1.08,1.49) | 0.003 |  |
| AKT2 | rs16974157 | CA vs. CC | 7211/20937 | 473/1366 | 0.99 | (0.85,1.16) | 0.88 |  | 8287/21489 | 340/1042 | 0.84 | (0.71,0.99) | 0.04 |  |
|  |  | AA vs. CC | 661/20937 | 37/1366 | 0.85 | (0.54,1.34) | 0.48 |  | 601/21489 | 26/1042 | 0.94 | (0.55,1.61) | 0.83 |  |
|  |  | Per minor allele | 28808 | 1876 | 0.97 | (0.85,1.11) | 0.64 |  | 30377 | 1408 | 0.87 | (0.75,1.01) | 0.06 |  |
|  | rs7250897 | CT vs. CC | 11863/14353 | 793/924 | 1.02 | (0.89,1.18) | 0.74 |  | 12379/15159 | 596/690 | 1.06 | (0.92,1.24) | 0.42 |  |
|  |  | TT vs. CC | 2605/14353 | 165/924 | 1.01 | (0.80,1.29) | 0.92 |  | 2852/15159 | 125/690 | 0.97 | (0.75,1.25) | 0.79 |  |
|  |  | Per minor allele | 28821 | 1882 | 1.01 | (0.91,1.12) | 0.80 |  | 30390 | 1411 | 1.01 | (0.91,1.13) | 0.80 |  |
|  | rs874269 | CT vs. CC | 4131/24496 | 250/1619 | 0.91 | (0.75,1.10) | 0.32 |  | 4532/25711 | 227/1173 | 1.09 | (0.89,1.33) | 0.39 |  |
|  |  | TT vs. CC | 214/24496 | 13/1619 | 1.01 | (0.46,2.25) | 0.97 |  | 154/25711 | 11/1173 | 1.81 | (0.73,4.50) | 0.20 |  |
|  |  | Per minor allele | 28841 | 1882 | 0.92 | (0.77,1.10) | 0.38 |  | 30397 | 1411 | 1.13 | (0.93,1.36) | 0.21 |  |
| IGF1R (from GWAS) | rs2871865 | CG vs. CC | 5244/23333 | 333/1533 | 0.96 | (0.80,1.14) | 0.62 |  | 5379/24512 | 255/1137 | 1.02 | (0.85,1.23) | 0.80 |  |
|  |  | GG vs. CC | 264/23333 | 16/1533 | 1.02 | (0.50,2.06) | 0.96 |  | 506/24512 | 18/1137 | 0.78 | (0.43,1.41) | 0.41 |  |
|  |  | Per minor allele | 28841 | 1882 | 0.97 | (0.82,1.13) | 0.67 |  | 30397 | 1410 | 0.98 | (0.84,1.15) | 0.85 |  |
| INSR (from GWAS) | rs891088 | AG vs. AA | 10861/15404 | 678/1047 | 0.91 | (0.79,1.05) | 0.18 |  | 11527/16821 | 557/751 | 1.07 | (0.92,1.25) | 0.37 |  |
|  |  | GG vs. AA | 2077/15404 | 144/1047 | 1.00 | (0.77,1.30) | 1.00 |  | 1612/16821 | 96/751 | 1.40 | (1.03,1.90) | 0.03 |  |
|  |  | Per minor allele | 28343 | 1869 | 0.96 | (0.86,1.07) | 0.43 |  | 29960 | 1404 | 1.13 | (1.00,1.27) | 0.05 |  |
| RICTOR (from GWAS) | rs2043112 | GA vs. GG | 14395/9172 | 894/639 | 0.90 | (0.77,1.04) | 0.16 |  | 14686/10303 | 669/477 | 0.98 | (0.83,1.15) | 0.77 |  |
|  |  | AA vs. GG | 5275/9172 | 348/639 | 0.95 | (0.78,1.16) | 0.63 |  | 5388/10303 | 265/477 | 1.05 | (0.85,1.30) | 0.64 |  |
|  |  | Per minor allele | 28841 | 1881 | 0.96 | (0.87,1.06) | 0.46 |  | 30377 | 1411 | 1.02 | (0.92,1.13) | 0.73 |  |
| RPTOR (from GWAS) | rs7503807 | AC vs. AA | 13394/9573 | 948/584 | 1.17 | (1.01,1.37) | 0.04 |  | 15049/9515 | 724/419 | 1.10 | (0.94,1.30) | 0.25 |  |
|  |  | CC vs. AA | 5848/9573 | 350/584 | 0.98 | (0.81,1.18) | 0.81 |  | 5813/9515 | 266/419 | 1.04 | (0.84,1.28) | 0.74 |  |
|  |  | Per minor allele | 28815 | 1882 | 1.01 | (0.92,1.11) | 0.85 |  | 30377 | 1409 | 1.03 | (0.93,1.14) | 0.61 |  |
| S6K1 (from GWAS) | rs1051424 | AG vs. AA | 1693/25395 | 102/1647 | 0.94 | (0.70,1.25) | 0.67 |  | 1564/26692 | 72/1237 | 0.98 | (0.71,1.36) | 0.92 |  |
|  |  | GG vs. AA | 1160/25395 | 66/1647 | 0.87 | (0.62,1.24) | 0.45 |  | 877/26692 | 45/1237 | 1.14 | (0.75,1.73) | 0.55 |  |
|  |  | Per minor allele | 28248 | 1815 | 0.94 | (0.80,1.09) | 0.40 |  | 29132 | 1354 | 1.04 | (0.87,1.25) | 0.66 |  |
| ^a^ Age-adjusted hazard ratio for colorectal cancer.  ^b^ Gene-based false discovery rate-adjusted *P* value according to the Benjamini-Hochberg criterium, using 0.20 as the false discovery rate threshold. | | | | | | | | | | | | | | |

| **Supplemental Table 5.** Associations between exposures related to energy balance and colon cancer risk in men and women, stratified for tertiles of the polygenic risk score of mTOR-PI3K-Akt pathway polymorphisms in the Netherlands Cohort Study (20.3 years of follow-up) | | | | | | | | | | | | | | | | | | | | | |
| --- | --- | --- | --- | --- | --- | --- | --- | --- | --- | --- | --- | --- | --- | --- | --- | --- | --- | --- | --- | --- | --- |
|  |  | **Men** | | | | | | | | | | **Women** | | | | | | | | | |
|  |  | **BMI** | | | | | | | | | | **BMI** | | | | | | | | | |
|  |  | **T1 (sex-specific)** | | | **T2 (sex-specific)** | | | **T3 (sex-specific)** | | |  | **T1 (sex-specific)** | | | **T2 (sex-specific)** | | | **T3 (sex-specific)** | | |  |
|  |  | **N cases/PT at risk** | **HR^a,b^** | **(95% CI)** | **N cases/PT at risk** | **HR^a,b^** | **(95% CI)** | **N cases/PT at risk** | **HR^a,b^** | **(95% CI)** | **P for interaction** | **N cases/PT at risk** | **HR^a,b^** | **(95% CI)** | **N cases/PT at risk** | **HR^a,b^** | **(95% CI)** | **N cases/PT at risk** | **HR^a,b^** | **(95% CI)** | **P for interaction** |
| **Polygenic** **risk score** | **T1** | 111/3343 | 1.00 | (ref.) | 103/2911 | 0.96 | (0.68,1.38) | 127/2588 | 1.34 | (0.94,1.91) |  | 107/3207 | 1.00 | (ref.) | 103/2999 | 1.04 | (0.72,1.49) | 83/2788 | 0.89 | (0.60,1.30) |  |
|  | **T2** | 105/2907 | 1.00 | (ref.) | 127/3196 | 1.05 | (0.74,1.50) | 133/2810 | 1.26 | (0.86,1.83) |  | 107/2829 | 1.00 | (ref.) | 99/2822 | 0.97 | (0.66,1.41) | 71/2775 | 0.71 | (0.47,1.08) |  |
|  | **T3** | 126/2688 | 1.00 | (ref.) | 129/3046 | 0.89 | (0.63,1.26) | 149/2726 | 1.09 | (0.77,1.56) | 0.63 | 115/3000 | 1.00 | (ref.) | 103/2900 | 0.89 | (0.63,1.27) | 124/2746 | 1.17 | (0.82,1.67) | 0.13 |
|  |  | **Trouser/skirt size** | | | | | | | | | | **Trouser/skirt size** | | | | | | | | | |
|  |  | **≤median (sex-specific)** | | | **>median (sex-specific)** | | |  | | |  | **≤median (sex-specific)** | | | **>median (sex-specific)** | | |  | | |  |
|  |  | **N cases/PT at risk** | **HR^a,c^** | **(95% CI)** | **N cases/PT at risk** | **HR^a,c^** | **(95% CI)** | **N cases/PT at risk** | **HR^a,c^** | **(95% CI)** | **P for interaction** | **N cases/PT at risk** | **HR^a,c^** | **(95% CI)** | **N cases/PT at risk** | **HR^a,c^** | **(95% CI)** | **N cases/PT at risk** | **HR^a,c^** | **(95% CI)** | **P for interaction** |
| **Polygenic** **risk score** | **T1** | 104/3250 | 1.00 | (ref.) | 207/5042 | 1.15 | (0.79,1.67) |  |  |  |  | 126/3967 | 1.00 | (ref.) | 164/4910 | 1.08 | (0.72,1.61) |  |  |  |  |
|  | **T2** | 110/2932 | 1.00 | (ref.) | 226/5019 | 1.14 | (0.80,1.62) |  |  |  |  | 137/3849 | 1.00 | (ref.) | 135/4437 | 0.91 | (0.60,1.40) |  |  |  |  |
|  | **T3** | 111/3062 | 1.00 | (ref.) | 254/4775 | 1.36 | (0.96,1.93) |  |  |  | 0.66 | 136/3628 | 1.00 | (ref.) | 204/4937 | 1.08 | (0.75,1.56) |  |  |  | 0.69 |
|  |  | **Non-occupational physical activity** | | | | | | | | | | **Non-occupational physical activity** | | | | | | | | | |
|  |  | **≤30 min/day** | | | **>30-60 min/day** | | | **>60 min/day** | | |  | **≤30 min/day** | | | **>30-60 min/day** | | | **>60 min/day** | | |  |
|  |  | **N cases/PT at risk** | **HR^a,d^** | **(95% CI)** | **N cases/PT at risk** | **HR^a,d^** | **(95% CI)** | **N cases/PT at risk** | **HR^a,d^** | **(95% CI)** | **P for interaction** | **N cases/PT at risk** | **HR^a,d^** | **(95% CI)** | **N cases/PT at risk** | **HR^a,d^** | **(95% CI)** | **N cases/PT at risk** | **HR^a,d^** | **(95% CI)** | **P for interaction** |
| **Polygenic** **risk score** | **T1** | 56/1380 | 1.00 | (ref.) | 95/3011 | 0.76 | (0.49,1.18) | 190/4452 | 1.05 | (0.70,1.59) |  | 85/1887 | 1.00 | (ref.) | 75/2964 | 0.54 | (0.35,0.83) | 133/4142 | 0.70 | (0.48,1.02) |  |
|  | **T2** | 53/1378 | 1.00 | (ref.) | 110/2811 | 1.06 | (0.68,1.66) | 202/4724 | 1.14 | (0.76,1.71) |  | 72/1775 | 1.00 | (ref.) | 93/2790 | 0.82 | (0.54,1.26) | 112/3860 | 0.71 | (0.48,1.07) |  |
|  | **T3** | 64/1341 | 1.00 | (ref.) | 132/2766 | 0.88 | (0.57,1.36) | 208/4353 | 0.87 | (0.58,1.30) | 0.76 | 71/1933 | 1.00 | (ref.) | 128/2815 | 1.31 | (0.88,1.96) | 143/3898 | 1.02 | (0.70,1.49) | 0.46 |
|  |  | **Height,**  **T1 (sex-specific)** | | | **Height,**  **T2 (sex-specific)** | | | **Height,**  **T3 (sex-specific)** | | |  | **Height,**  **T1 (sex-specific)** | | | **Height,**  **T2 (sex-specific)** | | | **Height,**  **T3 (sex-specific)** | | |  |
|  |  | **N cases/PT at risk** | **HR^a,c^** | **(95% CI)** | **N cases/PT at risk** | **HR^a,c^** | **(95% CI)** | **N cases/PT at risk** | **HR^a,c^** | **(95% CI)** | **P for interaction** | **N cases/PT at risk** | **HR^a,c^** | **(95% CI)** | **N cases/PT at risk** | **HR^a,c^** | **(95% CI)** | **N cases/PT at risk** | **HR^a,c^** | **(95% CI)** | **P for interaction** |
| **Polygenic** **risk score** | **T1** | 93/3074 | 1.00 | (ref.) | 121/2862 | 1.54 | (1.07,2.23) | 127/2906 | 1.61 | (1.12,2.32) |  | 90/3605 | 1.00 | (ref.) | 102/3156 | 1.32 | (0.91,1.92) | 101/2233 | 2.02 | (1.36,2.99) |  |
|  | **T2** | 114/3205 | 1.00 | (ref.) | 116/2616 | 1.44 | (1.00,2.09) | 135/3093 | 1.45 | (1.02,2.07) |  | 95/3130 | 1.00 | (ref.) | 96/2905 | 1.04 | (0.71,1.52) | 86/2390 | 1.06 | (0.70,1.60) |  |
|  | **T3** | 113/2826 | 1.00 | (ref.) | 130/2987 | 1.14 | (0.80,1.62) | 161/2647 | 1.50 | (1.06,2.13) | 0.84 | 117/3285 | 1.00 | (ref.) | 121/2761 | 1.43 | (0.99,2.08) | 104/2600 | 1.36 | (0.91,2.02) | 0.11 |
|  |  | **Exposure to energy restriction during childhood and adolescence as based on place of residence during the Hunger Winter** | | | | | | | | | | **Exposure to energy restriction during childhood and adolescence as based on place of residence during the Hunger Winter** | | | | | | | | | |
|  |  | **Non-Western area** | | | **Western rural area** | | | **Western city** | | |  | **Non-Western area** | | | **Western rural area** | | | **Western city** | | |  |
|  |  | **N cases/PT at risk** | **HR^a,c^** | **(95% CI)** | **N cases/PT at risk** | **HR^a,c^** | **(95% CI)** | **N cases/PT at risk** | **HR^a,c^** | **(95% CI)** | **P for interaction** | **N cases/PT at risk** | **HR^a,c^** | **(95% CI)** | **N cases/PT at risk** | **HR^a,c^** | **(95% CI)** | **N cases/PT at risk** | **HR^a,c^** | **(95% CI)** | **P for interaction** |
| **Polygenic** **risk score** | **T1** | 170/4418 | 1.00 | (ref.) | 42/1127 | 1.12 | (0.70,1.80) | 75/1918 | 1.10 | (0.75,1.62) |  | 148/4989 | 1.00 | (ref.) | 48/1182 | 1.60 | (1.00,2.56) | 80/2291 | 1.30 | (0.89,1.90) |  |
|  | **T2** | 185/4307 | 1.00 | (ref.) | 47/1230 | 0.97 | (0.62,1.53) | 71/1696 | 0.86 | (0.58,1.27) |  | 139/4413 | 1.00 | (ref.) | 44/1222 | 1.13 | (0.71,1.81) | 79/2333 | 1.12 | (0.77,1.62) |  |
|  | **T3** | 205/4194 | 1.00 | (ref.) | 50/946 | 1.26 | (0.80,2.00) | 77/1805 | 0.82 | (0.55,1.20) | 0.60 | 167/4412 | 1.00 | (ref.) | 58/1283 | 1.22 | (0.80,1.88) | 101/2462 | 1.11 | (0.78,1.57) | 0.82 |
| Abbreviations: BMI, body mass index; CI, confidence interval; HR, hazard ratio; N, number of; PT, person-time; ref., reference; T1-3, tertile 1-3.  ^a^ Adjusted for age (years), first-degree family history of colorectal cancer (yes/no); smoking status (never, ex, current); alcohol intake (0, 0.1-29, ≥30 g/d); meat intake (g/d), processed meat intake (g/d), and total energy intake (kcal/d).  ^b^ Additionally adjusted for non-occupational physical activity (≤30, >30-60, >60 min/day), respectively.  ^c^ Additionally adjusted for BMI (kg/m^2^) and non-occupational physical activity (≤30, >30-60, >60 min/day), respectively.  ^d^ Additionally adjusted for BMI (kg/m^2^). | | | | | | | | | | | | | | | | | | | | | |

| **Supplemental Table 6.** Associations between exposures related to energy balance and proximal colon cancer risk in men and women, stratified for tertiles of the polygenic risk score of mTOR-PI3K-Akt pathway polymorphisms in the Netherlands Cohort Study (20.3 years of follow-up) | | | | | | | | | | | | | | | | | | | | | |
| --- | --- | --- | --- | --- | --- | --- | --- | --- | --- | --- | --- | --- | --- | --- | --- | --- | --- | --- | --- | --- | --- |
|  |  | **Men** | | | | | | | | | | **Women** | | | | | | | | | |
|  |  | **BMI** | | | | | | | | | | **BMI** | | | | | | | | | |
|  |  | **T1 (sex-specific)** | | | **T2 (sex-specific)** | | | **T3 (sex-specific)** | | |  | **T1 (sex-specific)** | | | **T2 (sex-specific)** | | | **T3 (sex-specific)** | | |  |
|  |  | **N cases/PT at risk** | **HR^a,b^** | **(95% CI)** | **N cases/PT at risk** | **HR^a,b^** | **(95% CI)** | **N cases/PT at risk** | **HR^a,b^** | **(95% CI)** | **P for interaction** | **N cases/PT at risk** | **HR^a,b^** | **(95% CI)** | **N cases/PT at risk** | **HR^a,b^** | **(95% CI)** | **N cases/PT at risk** | **HR^a,b^** | **(95% CI)** | **P for interaction** |
| **Polygenic** **risk score** | **T1** | 48/3343 | 1.00 | (ref.) | 47/2911 | 1.02 | (0.63,1.64) | 65/2588 | 1.59 | (1.00,2.53) |  | 61/3207 | 1.00 | (ref.) | 62/2999 | 1.14 | (0.74,1.77) | 47/2788 | 0.94 | (0.58,1.51) |  |
|  | **T2** | 44/2907 | 1.00 | (ref.) | 67/3196 | 1.34 | (0.84,2.11) | 54/2810 | 1.26 | (0.76,2.08) |  | 68/2829 | 1.00 | (ref.) | 61/2822 | 0.96 | (0.62,1.49) | 38/2775 | 0.61 | (0.37,1.01) |  |
|  | **T3** | 61/2688 | 1.00 | (ref.) | 56/3046 | 0.79 | (0.50,1.23) | 74/2726 | 1.11 | (0.72,1.73) | 0.44 | 71/3000 | 1.00 | (ref.) | 64/2900 | 0.88 | (0.58,1.34) | 68/2746 | 1.05 | (0.69,1.60) | 0.20 |
|  |  | **Trouser/skirt size** | | | | | | | | | | **Trouser/skirt size** | | | | | | | | | |
|  |  | **≤median (sex-specific)** | | | **>median (sex-specific)** | | |  | | |  | **≤median (sex-specific)** | | | **>median (sex-specific)** | | |  | | |  |
|  |  | **N cases/PT at risk** | **HR^a,c^** | **(95% CI)** | **N cases/PT at risk** | **HR^a,c^** | **(95% CI)** | **N cases/PT at risk** | **HR^a,c^** | **(95% CI)** | **P for interaction** | **N cases/PT at risk** | **HR^a,c^** | **(95% CI)** | **N cases/PT at risk** | **HR^a,c^** | **(95% CI)** | **N cases/PT at risk** | **HR^a,c^** | **(95% CI)** | **P for interaction** |
| **Polygenic** **risk score** | **T1** | 43/3250 | 1.00 | (ref.) | 101/5042 | 1.29 | (0.77,2.14) |  |  |  |  | 74/3967 | 1.00 | (ref.) | 94/4910 | 0.99 | (0.61,1.61) |  |  |  |  |
|  | **T2** | 56/2932 | 1.00 | (ref.) | 96/5019 | 0.94 | (0.59,1.50) |  |  |  |  | 90/3849 | 1.00 | (ref.) | 75/4437 | 0.75 | (0.45,1.27) |  |  |  |  |
|  | **T3** | 54/3062 | 1.00 | (ref.) | 118/4775 | 1.32 | (0.85,2.05) |  |  |  | 0.73 | 81/3628 | 1.00 | (ref.) | 121/4937 | 1.03 | (0.66,1.59) |  |  |  | 0.60 |
|  |  | **Non-occupational physical activity** | | | | | | | | | | **Non-occupational physical activity** | | | | | | | | | |
|  |  | **≤30 min/day** | | | **>30-60 min/day** | | | **>60 min/day** | | |  | **≤30 min/day** | | | **>30-60 min/day** | | | **>60 min/day** | | |  |
|  |  | **N cases/PT at risk** | **HR^a,d^** | **(95% CI)** | **N cases/PT at risk** | **HR^a,d^** | **(95% CI)** | **N cases/PT at risk** | **HR^a,d^** | **(95% CI)** | **P for interaction** | **N cases/PT at risk** | **HR^a,d^** | **(95% CI)** | **N cases/PT at risk** | **HR^a,d^** | **(95% CI)** | **N cases/PT at risk** | **HR^a,d^** | **(95% CI)** | **P for interaction** |
| **Polygenic** **risk score** | **T1** | 31/1380 | 1.00 | (ref.) | 43/3011 | 0.60 | (0.35,1.05) | 86/4452 | 0.86 | (0.52,1.44) |  | 47/1887 | 1.00 | (ref.) | 45/2964 | 0.58 | (0.35,0.98) | 78/4142 | 0.75 | (0.48,1.19) |  |
|  | **T2** | 28/1378 | 1.00 | (ref.) | 51/2811 | 0.96 | (0.55,1.69) | 86/4724 | 0.93 | (0.56,1.56) |  | 39/1775 | 1.00 | (ref.) | 55/2790 | 0.90 | (0.53,1.50) | 73/3860 | 0.85 | (0.53,1.36) |  |
|  | **T3** | 29/1341 | 1.00 | (ref.) | 68/2766 | 1.05 | (0.61,1.83) | 94/4353 | 0.90 | (0.54,1.53) | 0.97 | 41/1933 | 1.00 | (ref.) | 79/2815 | 1.46 | (0.91,2.34) | 83/3898 | 1.04 | (0.66,1.63) | 0.76 |
|  |  | **Height,**  **T1 (sex-specific)** | | | **Height,**  **T2 (sex-specific)** | | | **Height,**  **T3 (sex-specific)** | | |  | **Height,**  **T1 (sex-specific)** | | | **Height,**  **T2 (sex-specific)** | | | **Height,**  **T3 (sex-specific)** | | |  |
|  |  | **N cases/PT at risk** | **HR^a,c^** | **(95% CI)** | **N cases/PT at risk** | **HR^a,c^** | **(95% CI)** | **N cases/PT at risk** | **HR^a,c^** | **(95% CI)** | **P for interaction** | **N cases/PT at risk** | **HR^a,c^** | **(95% CI)** | **N cases/PT at risk** | **HR^a,c^** | **(95% CI)** | **N cases/PT at risk** | **HR^a,c^** | **(95% CI)** | **P for interaction** |
| **Polygenic** **risk score** | **T1** | 45/3074 | 1.00 | (ref.) | 50/2862 | 1.35 | (0.83,2.18) | 65/2906 | 1.73 | (1.09,2.74) |  | 52/3605 | 1.00 | (ref.) | 62/3156 | 1.37 | (0.87,2.17) | 56/2233 | 1.95 | (1.20,3.16) |  |
|  | **T2** | 52/3205 | 1.00 | (ref.) | 51/2616 | 1.41 | (0.87,2.30) | 62/3093 | 1.50 | (0.95,2.38) |  | 58/3130 | 1.00 | (ref.) | 63/2905 | 1.16 | (0.73,1.85) | 46/2390 | 0.91 | (0.55,1.50) |  |
|  | **T3** | 65/2826 | 1.00 | (ref.) | 54/2987 | 0.81 | (0.51,1.26) | 72/2647 | 1.17 | (0.76,1.79) | 0.61 | 65/3285 | 1.00 | (ref.) | 77/2761 | 1.69 | (1.09,2.63) | 61/2600 | 1.49 | (0.93,2.38) | 0.19 |
|  |  | **Exposure to energy restriction during childhood and adolescence as based on place of residence during the Hunger Winter** | | | | | | | | | | **Exposure to energy restriction during childhood and adolescence as based on place of residence during the Hunger Winter** | | | | | | | | | |
|  |  | **Non-Western area** | | | **Western rural area** | | | **Western city** | | |  | **Non-Western area** | | | **Western rural area** | | | **Western city** | | |  |
|  |  | **N cases/PT at risk** | **HR^a,c^** | **(95% CI)** | **N cases/PT at risk** | **HR^a,c^** | **(95% CI)** | **N cases/PT at risk** | **HR^a,c^** | **(95% CI)** | **P for interaction** | **N cases/PT at risk** | **HR^a,c^** | **(95% CI)** | **N cases/PT at risk** | **HR^a,c^** | **(95% CI)** | **N cases/PT at risk** | **HR^a,c^** | **(95% CI)** | **P for interaction** |
| **Polygenic** **risk score** | **T1** | 85/4418 | 1.00 | (ref.) | 21/1127 | 1.15 | (0.63,2.09) | 25/1918 | 0.74 | (0.43,1.27) |  | 78/4989 | 1.00 | (ref.) | 32/1182 | 2.16 | (1.23,3.81) | 51/2291 | 1.64 | (1.02,2.63) |  |
|  | **T2** | 79/4307 | 1.00 | (ref.) | 22/1230 | 1.12 | (0.62,2.04) | 36/1696 | 1.03 | (0.63,1.68) |  | 78/4413 | 1.00 | (ref.) | 23/1222 | 1.06 | (0.60,1.89) | 56/2333 | 1.36 | (0.88,2.10) |  |
|  | **T3** | 112/4194 | 1.00 | (ref.) | 26/946 | 1.24 | (0.71,2.16) | 24/1805 | 0.46 | (0.27,0.80) | 0.06 | 94/4412 | 1.00 | (ref.) | 37/1283 | 1.40 | (0.85,2.30) | 61/2462 | 1.19 | (0.79,1.79) | 0.63 |
| Abbreviations: BMI, body mass index; CI, confidence interval; HR, hazard ratio; N, number of; PT, person-time; ref., reference; T1-3, tertile 1-3.  ^a^ Adjusted for age (years), first-degree family history of colorectal cancer (yes/no); smoking status (never, ex, current); alcohol intake (0, 0.1-29, ≥30 g/d); meat intake (g/d), processed meat intake (g/d), and total energy intake (kcal/d).  ^b^ Additionally adjusted for non-occupational physical activity (≤30, >30-60, >60 min/day), respectively.  ^c^ Additionally adjusted for BMI (kg/m^2^) and non-occupational physical activity (≤30, >30-60, >60 min/day), respectively.  ^d^ Additionally adjusted for BMI (kg/m^2^). | | | | | | | | | | | | | | | | | | | | | |

| **Supplemental Table 7.** Associations between exposures related to energy balance and distal colon cancer risk in men and women, stratified for tertiles of the polygenic risk score of mTOR/PI3K/Akt pathway polymorphisms in the Netherlands Cohort Study (20.3 years of follow-up) | | | | | | | | | | | | | | | | | | | | | |
| --- | --- | --- | --- | --- | --- | --- | --- | --- | --- | --- | --- | --- | --- | --- | --- | --- | --- | --- | --- | --- | --- |
|  |  | **Men** | | | | | | | | | | **Women** | | | | | | | | | |
|  |  | **BMI** | | | | | | | | | | **BMI** | | | | | | | | | |
|  |  | **T1 (sex-specific)** | | | **T2 (sex-specific)** | | | **T3 (sex-specific)** | | |  | **T1 (sex-specific)** | | | **T2 (sex-specific)** | | | **T3 (sex-specific)** | | |  |
|  |  | **N cases/PT at risk** | **HR^a,b^** | **(95% CI)** | **N cases/PT at risk** | **HR^a,b^** | **(95% CI)** | **N cases/PT at risk** | **HR^a,b^** | **(95% CI)** | **P for interaction** | **N cases/PT at risk** | **HR^a,b^** | **(95% CI)** | **N cases/PT at risk** | **HR^a,b^** | **(95% CI)** | **N cases/PT at risk** | **HR^a,b^** | **(95% CI)** | **P for interaction** |
| **Polygenic** **risk score** | **T1** | 60/3343 | 1.00 | (ref.) | 50/2911 | 0.86 | (0.55,1.34) | 61/2588 | 1.17 | (0.75,1.81) |  | 44/3207 | 1.00 | (ref.) | 38/2999 | 0.89 | (0.54,1.48) | 34/2788 | 0.82 | (0.49,1.38) |  |
|  | **T2** | 58/2907 | 1.00 | (ref.) | 53/3196 | 0.79 | (0.50,1.23) | 75/2810 | 1.25 | (0.79,1.98) |  | 35/2829 | 1.00 | (ref.) | 37/2822 | 1.08 | (0.64,1.82) | 31/2775 | 0.92 | (0.51,1.66) |  |
|  | **T3** | 61/2688 | 1.00 | (ref.) | 70/3046 | 1.01 | (0.65,1.57) | 74/2726 | 1.14 | (0.72,1.80) | 0.88 | 40/3000 | 1.00 | (ref.) | 38/2900 | 0.95 | (0.58,1.58) | 52/2746 | 1.38 | (0.85,2.23) | 0.29 |
|  |  | **Trouser/skirt size** | | | | | | | | | | **Trouser/skirt size** | | | | | | | | | |
|  |  | **≤median (sex-specific)** | | | **>median (sex-specific)** | | |  | | |  | **≤median (sex-specific)** | | | **>median (sex-specific)** | | |  | | |  |
|  |  | **N cases/PT at risk** | **HR^a,c^** | **(95% CI)** | **N cases/PT at risk** | **HR^a,c^** | **(95% CI)** | **N cases/PT at risk** | **HR^a,c^** | **(95% CI)** | **P for interaction** | **N cases/PT at risk** | **HR^a,c^** | **(95% CI)** | **N cases/PT at risk** | **HR^a,c^** | **(95% CI)** | **N cases/PT at risk** | **HR^a,c^** | **(95% CI)** | **P for interaction** |
| **Polygenic** **risk score** | **T1** | 57/3250 | 1.00 | (ref.) | 100/5042 | 1.07 | (0.67,1.71) |  |  |  |  | 50/3967 | 1.00 | (ref.) | 65/4910 | 1.16 | (0.66,2.05) |  |  |  |  |
|  | **T2** | 53/2932 | 1.00 | (ref.) | 119/5019 | 1.22 | (0.80,1.87) |  |  |  |  | 43/3849 | 1.00 | (ref.) | 57/4437 | 1.21 | (0.66,2.23) |  |  |  |  |
|  | **T3** | 54/3062 | 1.00 | (ref.) | 133/4775 | 1.43 | (0.93,2.22) |  |  |  | 0.30 | 51/3628 | 1.00 | (ref.) | 78/4937 | 1.16 | (0.71,1.89) |  |  |  | 0.85 |
|  |  | **Non-occupational physical activity** | | | | | | | | | | **Non-occupational physical activity** | | | | | | | | | |
|  |  | **≤30 min/day** | | | **>30-60 min/day** | | | **>60 min/day** | | |  | **≤30 min/day** | | | **>30-60 min/day** | | | **>60 min/day** | | |  |
|  |  | **N cases/PT at risk** | **HR^a,d^** | **(95% CI)** | **N cases/PT at risk** | **HR^a,d^** | **(95% CI)** | **N cases/PT at risk** | **HR^a,d^** | **(95% CI)** | **P for interaction** | **N cases/PT at risk** | **HR^a,d^** | **(95% CI)** | **N cases/PT at risk** | **HR^a,d^** | **(95% CI)** | **N cases/PT at risk** | **HR^a,d^** | **(95% CI)** | **P for interaction** |
| **Polygenic** **risk score** | **T1** | 25/1380 | 1.00 | (ref.) | 48/3011 | 0.87 | (0.50,1.53) | 98/4452 | 1.22 | (0.73,2.04) |  | 36/1887 | 1.00 | (ref.) | 28/2964 | 0.48 | (0.26,0.86) | 52/4142 | 0.62 | (0.38,1.03) |  |
|  | **T2** | 23/1378 | 1.00 | (ref.) | 53/2811 | 1.14 | (0.63,2.05) | 110/4724 | 1.39 | (0.82,2.37) |  | 32/1775 | 1.00 | (ref.) | 37/2790 | 0.73 | (0.41,1.29) | 34/3860 | 0.49 | (0.28,0.86) |  |
|  | **T3** | 32/1341 | 1.00 | (ref.) | 62/2766 | 0.79 | (0.46,1.36) | 111/4353 | 0.89 | (0.54,1.48) | 0.52 | 27/1933 | 1.00 | (ref.) | 47/2815 | 1.20 | (0.69,2.09) | 56/3898 | 1.05 | (0.62,1.77) | 0.16 |
|  |  | **Height,**  **T1 (sex-specific)** | | | **Height,**  **T2 (sex-specific)** | | | **Height,**  **T3 (sex-specific)** | | |  | **Height,**  **T1 (sex-specific)** | | | **Height,**  **T2 (sex-specific)** | | | **Height,**  **T3 (sex-specific)** | | |  |
|  |  | **N cases/PT at risk** | **HR^a,c^** | **(95% CI)** | **N cases/PT at risk** | **HR^a,c^** | **(95% CI)** | **N cases/PT at risk** | **HR^a,c^** | **(95% CI)** | **P for interaction** | **N cases/PT at risk** | **HR^a,c^** | **(95% CI)** | **N cases/PT at risk** | **HR^a,c^** | **(95% CI)** | **N cases/PT at risk** | **HR^a,c^** | **(95% CI)** | **P for interaction** |
| **Polygenic** **risk score** | **T1** | 43/3074 | 1.00 | (ref.) | 69/2862 | 1.89 | (1.20,2.97) | 59/2906 | 1.61 | (1.01,2.56) |  | 35/3605 | 1.00 | (ref.) | 39/3156 | 1.32 | (0.79,2.22) | 42/2233 | 2.13 | (1.25,3.63) |  |
|  | **T2** | 61/3205 | 1.00 | (ref.) | 61/2616 | 1.40 | (0.88,2.20) | 64/3093 | 1.25 | (0.81,1.94) |  | 35/3130 | 1.00 | (ref.) | 33/2905 | 0.92 | (0.53,1.58) | 35/2390 | 1.22 | (0.68,2.19) |  |
|  | **T3** | 47/2826 | 1.00 | (ref.) | 72/2987 | 1.56 | (0.99,2.48) | 86/2647 | 1.93 | (1.22,3.05) | 0.27 | 49/3285 | 1.00 | (ref.) | 41/2761 | 1.11 | (0.66,1.87) | 40/2600 | 1.18 | (0.68,2.05) | 0.18 |
|  |  | **Exposure to energy restriction during childhood and adolescence as based on place of residence during the Hunger Winter** | | | | | | | | | | **Exposure to energy restriction during childhood and adolescence as based on place of residence during the Hunger Winter** | | | | | | | | | |
|  |  | **Non-Western area** | | | **Western rural area** | | | **Western city** | | |  | **Non-Western area** | | | **Western rural area** | | | **Western city** | | |  |
|  |  | **N cases/PT at risk** | **HR^a,c^** | **(95% CI)** | **N cases/PT at risk** | **HR^a,c^** | **(95% CI)** | **N cases/PT at risk** | **HR^a,c^** | **(95% CI)** | **P for interaction** | **N cases/PT at risk** | **HR^a,c^** | **(95% CI)** | **N cases/PT at risk** | **HR^a,c^** | **(95% CI)** | **N cases/PT at risk** | **HR^a,c^** | **(95% CI)** | **P for interaction** |
| **Polygenic** **risk score** | **T1** | 82/4418 | 1.00 | (ref.) | 20/1127 | 1.07 | (0.58,1.98) | 46/1918 | 1.36 | (0.86,2.15) |  | 65/4989 | 1.00 | (ref.) | 15/1182 | 1.06 | (0.54,2.07) | 28/2291 | 1.01 | (0.60,1.70) |  |
|  | **T2** | 102/4307 | 1.00 | (ref.) | 25/1230 | 0.89 | (0.52,1.53) | 27/1696 | 0.59 | (0.36,0.98) |  | 58/4413 | 1.00 | (ref.) | 19/1222 | 1.11 | (0.60,2.06) | 21/2333 | 0.73 | (0.41,1.28) |  |
|  | **T3** | 91/4194 | 1.00 | (ref.) | 23/946 | 1.31 | (0.71,2.43) | 49/1805 | 1.21 | (0.75,1.94) | 0.04 | 67/4412 | 1.00 | (ref.) | 19/1283 | 0.96 | (0.52,1.77) | 40/2462 | 1.09 | (0.68,1.74) | 0.44 |
| Abbreviations: BMI, body mass index; CI, confidence interval; HR, hazard ratio; N, number of; PT, person-time; ref., reference; T1-3, tertile 1-3.  ^a^ Adjusted for age (years), first-degree family history of colorectal cancer (yes/no); smoking status (never, ex, current); alcohol intake (0, 0.1-29, ≥30 g/d); meat intake (g/d), processed meat intake (g/d), and total energy intake (kcal/d).  ^b^ Additionally adjusted for non-occupational physical activity (≤30, >30-60, >60 min/day), respectively.  ^c^ Additionally adjusted for BMI (kg/m^2^) and non-occupational physical activity (≤30, >30-60, >60 min/day), respectively.  ^d^ Additionally adjusted for BMI (kg/m^2^). | | | | | | | | | | | | | | | | | | | | | |

| **Supplemental Table 8.** Associations between exposures related to energy balance and rectal cancer risk in men and women, stratified for tertiles of the polygenic risk score of mTOR/PI3K/Akt pathway polymorphisms in the Netherlands Cohort Study (20.3 years of follow-up) | | | | | | | | | | | | | | | | | | | | | |
| --- | --- | --- | --- | --- | --- | --- | --- | --- | --- | --- | --- | --- | --- | --- | --- | --- | --- | --- | --- | --- | --- |
|  |  | **Men** | | | | | | | | | | **Women** | | | | | | | | | |
|  |  | **BMI** | | | | | | | | | | **BMI** | | | | | | | | | |
|  |  | **T1 (sex-specific)** | | | **T2 (sex-specific)** | | | **T3 (sex-specific)** | | |  | **T1 (sex-specific)** | | | **T2 (sex-specific)** | | | **T3 (sex-specific)** | | |  |
|  |  | **N cases/PT at risk** | **HR^a,b^** | **(95% CI)** | **N cases/PT at risk** | **HR^a,b^** | **(95% CI)** | **N cases/PT at risk** | **HR^a,b^** | **(95% CI)** | **P for interaction** | **N cases/PT at risk** | **HR^a,b^** | **(95% CI)** | **N cases/PT at risk** | **HR^a,b^** | **(95% CI)** | **N cases/PT at risk** | **HR^a,b^** | **(95% CI)** | **P for interaction** |
| **Polygenic** **risk score** | **T1** | 45/3343 | 1.00 | (ref.) | 46/2911 | 1.13 | (0.70,1.82) | 54/2588 | 1.47 | (0.90,2.40) |  | 23/3207 | 1.00 | (ref.) | 25/2999 | 1.18 | (0.63,2.22) | 21/2788 | 1.07 | (0.55,2.10) |  |
|  | **T2** | 42/2907 | 1.00 | (ref.) | 55/3196 | 1.24 | (0.77,1.98) | 48/2810 | 1.24 | (0.73,2.10) |  | 25/2829 | 1.00 | (ref.) | 20/2822 | 0.76 | (0.39,1.49) | 29/2775 | 1.02 | (0.53,1.97) |  |
|  | **T3** | 46/2688 | 1.00 | (ref.) | 53/3046 | 0.95 | (0.59,1.54) | 52/2726 | 0.97 | (0.59,1.59) | 0.54 | 31/3000 | 1.00 | (ref.) | 20/2900 | 0.64 | (0.35,1.19) | 22/2746 | 0.78 | (0.43,1.43) | 0.61 |
|  |  | **Trouser/skirt size** | | | | | | | | | | **Trouser/skirt size** | | | | | | | | | |
|  |  | **≤median (sex-specific)** | | | **>median (sex-specific)** | | |  | | |  | **≤median (sex-specific)** | | | **>median (sex-specific)** | | |  | | |  |
|  |  | **N cases/PT at risk** | **HR^a,c^** | **(95% CI)** | **N cases/PT at risk** | **HR^a,c^** | **(95% CI)** | **N cases/PT at risk** | **HR^a,c^** | **(95% CI)** | **P for interaction** | **N cases/PT at risk** | **HR^a,c^** | **(95% CI)** | **N cases/PT at risk** | **HR^a,c^** | **(95% CI)** | **N cases/PT at risk** | **HR^a,c^** | **(95% CI)** | **P for interaction** |
| **Polygenic** **risk score** | **T1** | 45/3250 | 1.00 | (ref.) | 82/5042 | 1.05 | (0.66,1.68) |  |  |  |  | 34/3967 | 1.00 | (ref.) | 34/4910 | 0.73 | (0.38,1.41) |  |  |  |  |
|  | **T2** | 52/2932 | 1.00 | (ref.) | 83/5019 | 0.97 | (0.60,1.57) |  |  |  |  | 26/3849 | 1.00 | (ref.) | 47/4437 | 1.48 | (0.73,3.01) |  |  |  |  |
|  | **T3** | 52/3062 | 1.00 | (ref.) | 89/4775 | 1.03 | (0.65,1.62) |  |  |  | 0.83 | 33/3628 | 1.00 | (ref.) | 38/4937 | 0.86 | (0.45,1.64) |  |  |  | 0.83 |
|  |  | **Non-occupational physical activity** | | | | | | | | | | **Non-occupational physical activity** | | | | | | | | | |
|  |  | **≤30 min/day** | | | **>30-60 min/day** | | | **>60 min/day** | | |  | **≤30 min/day** | | | **>30-60 min/day** | | | **>60 min/day** | | |  |
|  |  | **N cases/PT at risk** | **HR^a,d^** | **(95% CI)** | **N cases/PT at risk** | **HR^a,d^** | **(95% CI)** | **N cases/PT at risk** | **HR^a,d^** | **(95% CI)** | **P for interaction** | **N cases/PT at risk** | **HR^a,d^** | **(95% CI)** | **N cases/PT at risk** | **HR^a,d^** | **(95% CI)** | **N cases/PT at risk** | **HR^a,d^** | **(95% CI)** | **P for interaction** |
| **Polygenic** **risk score** | **T1** | 22/1380 | 1.00 | (ref.) | 46/3011 | 0.98 | (0.54,1.77) | 77/4452 | 1.11 | (0.64,1.94) |  | 21/1887 | 1.00 | (ref.) | 19/2964 | 0.58 | (0.29,1.18) | 29/4142 | 0.62 | (0.33,1.17) |  |
|  | **T2** | 17/1378 | 1.00 | (ref.) | 45/2811 | 1.42 | (0.75,2.69) | 83/4724 | 1.48 | (0.81,2.69) |  | 22/1775 | 1.00 | (ref.) | 24/2790 | 0.73 | (0.38,1.40) | 28/3860 | 0.60 | (0.31,1.14) |  |
|  | **T3** | 28/1341 | 1.00 | (ref.) | 34/2766 | 0.57 | (0.30,1.05) | 89/4353 | 0.90 | (0.52,1.55) | 0.57 | 24/1933 | 1.00 | (ref.) | 17/2815 | 0.51 | (0.26,1.00) | 32/3898 | 0.64 | (0.35,1.17) | 0.98 |
|  |  | **Height,**  **T1 (sex-specific)** | | | **Height,**  **T2 (sex-specific)** | | | **Height,**  **T3 (sex-specific)** | | |  | **Height,**  **T1 (sex-specific)** | | | **Height,**  **T2 (sex-specific)** | | | **Height,**  **T3 (sex-specific)** | | |  |
|  |  | **N cases/PT at risk** | **HR^a,c^** | **(95% CI)** | **N cases/PT at risk** | **HR^a,c^** | **(95% CI)** | **N cases/PT at risk** | **HR^a,c^** | **(95% CI)** | **P for interaction** | **N cases/PT at risk** | **HR^a,c^** | **(95% CI)** | **N cases/PT at risk** | **HR^a,c^** | **(95% CI)** | **N cases/PT at risk** | **HR^a,c^** | **(95% CI)** | **P for interaction** |
| **Polygenic** **risk score** | **T1** | 52/3074 | 1.00 | (ref.) | 49/2862 | 1.01 | (0.63,1.62) | 44/2906 | 0.89 | (0.56,1.42) |  | 23/3605 | 1.00 | (ref.) | 29/3156 | 1.51 | (0.82,2.80) | 17/2233 | 1.25 | (0.63,2.50) |  |
|  | **T2** | 52/3205 | 1.00 | (ref.) | 47/2616 | 1.27 | (0.78,2.08) | 46/3093 | 0.95 | (0.58,1.55) |  | 27/3130 | 1.00 | (ref.) | 23/2905 | 0.84 | (0.45,1.57) | 24/2390 | 1.21 | (0.62,2.35) |  |
|  | **T3** | 55/2826 | 1.00 | (ref.) | 51/2987 | 0.95 | (0.59,1.52) | 45/2647 | 0.85 | (0.52,1.38) | 0.96 | 12/3285 | 1.00 | (ref.) | 31/2761 | 3.62 | (1.68,7.79) | 30/2600 | 3.74 | (1.72,8.15) | 0.08 |
|  |  | **Exposure to energy restriction during childhood and adolescence as based on place of residence during the Hunger Winter** | | | | | | | | | | **Exposure to energy restriction during childhood and adolescence as based on place of residence during the Hunger Winter** | | | | | | | | | |
|  |  | **Non-Western area** | | | **Western rural area** | | | **Western city** | | |  | **Non-Western area** | | | **Western rural area** | | | **Western city** | | |  |
|  |  | **N cases/PT at risk** | **HR^a,c^** | **(95% CI)** | **N cases/PT at risk** | **HR^a,c^** | **(95% CI)** | **N cases/PT at risk** | **HR^a,c^** | **(95% CI)** | **P for interaction** | **N cases/PT at risk** | **HR^a,c^** | **(95% CI)** | **N cases/PT at risk** | **HR^a,c^** | **(95% CI)** | **N cases/PT at risk** | **HR^a,c^** | **(95% CI)** | **P for interaction** |
| **Polygenic** **risk score** | **T1** | 76/4418 | 1.00 | (ref.) | 26/1127 | 1.53 | (0.87,2.68) | 21/1918 | 0.66 | (0.38,1.15) |  | 35/4989 | 1.00 | (ref.) | 9/1182 | 1.14 | (0.50,2.61) | 21/2291 | 1.28 | (0.70,2.35) |  |
|  | **T2** | 86/4307 | 1.00 | (ref.) | 11/1230 | 0.48 | (0.23,0.99) | 27/1696 | 0.71 | (0.43,1.20) |  | 40/4413 | 1.00 | (ref.) | 13/1222 | 1.31 | (0.65,2.65) | 17/2333 | 0.82 | (0.44,1.53) |  |
|  | **T3** | 90/4194 | 1.00 | (ref.) | 18/946 | 0.99 | (0.53,1.88) | 26/1805 | 0.64 | (0.36,1.11) | 0.88 | 42/4412 | 1.00 | (ref.) | 9/1283 | 0.84 | (0.38,1.87) | 18/2462 | 0.80 | (0.43,1.49) | 0.43 |
| Abbreviations: BMI, body mass index; CI, confidence interval; HR, hazard ratio; N, number of; PT, person-time; ref., reference; T1-3, tertile 1-3.  ^a^ Adjusted for age (years), first-degree family history of colorectal cancer (yes/no); smoking status (never, ex, current); alcohol intake (0, 0.1-29, ≥30 g/d); meat intake (g/d), processed meat intake (g/d), and total energy intake (kcal/d).  ^b^ Additionally adjusted for non-occupational physical activity (≤30, >30-60, >60 min/day), respectively.  ^c^ Additionally adjusted for BMI (kg/m^2^) and non-occupational physical activity (≤30, >30-60, >60 min/day), respectively.  ^d^ Additionally adjusted for BMI (kg/m^2^). | | | | | | | | | | | | | | | | | | | | | |
